# Accelerating charge transfer via nonconjugated polyelectrolyte interlayers toward efficient versatile photoredox catalysis

**DOI:** 10.1038/s42004-021-00589-w

**Published:** 2021-10-22

**Authors:** Tao Li, Chuang Feng, Boon Kar Yap, Xuhui Zhu, Biquan Xiong, Zhicai He, Wai-Yeung Wong

**Affiliations:** 1 State Key Laboratory of Luminescent Materials and Devices, School of Material Science and Engineering, Institute of Polymer Optoelectronic Materials and Devices, South China University of Technology, Guangzhou, 510640 China; 2 Electronic and Communications Department, College of Engineering, Universiti Tenaga Nasional, Kajang, Selangor 43000 Malaysia; 3 Institute of Sustainable Energy, Universiti Tenaga Nasional, Kajang, Selangor 43000 Malaysia; 4 Department of Applied Biology and Chemical Technology, and Research Institute for Smart Energy, The Hong Kong Polytechnic University, Hung Hom, Hong Kong, China

**Keywords:** Renewable energy, Photocatalysis

## Abstract

One of the challenges for high-efficiency single-component-based photoredox catalysts is the low charge transfer and extraction due to the high recombination rate. Here, we demonstrate a strategy to precisely control the charge separation and transport efficiency of the catalytic host by introducing electron or hole extraction interlayers to improve the catalytic efficiency. We use simple and easily available non-conjugated polyelectrolytes (NCPs) (i.e., polyethyleneimine, PEI; poly(allylamine hydrochloride), PAH) to form interlayers, wherein such NCPs consist of the nonconjugated backbone with charge transporting functional groups. Taking CdS as examples, it is shown that although PEI and PAH are insulators and therefore do not have the ability to conduct electricity, they can form good electron or hole transport extraction layers due to the higher charge-transfer kinetics of pendant groups along the backbones, thereby greatly improving the charge transfer capability of CdS. Consequently, the resultant PEI-/PAH-functionalized nanocomposites exhibit significantly enhanced and versatile photoredox catalysis.

## Abstract (web summary)

Conjugated polymers are commonly used as charge conducting systems in photocatalysts, but the use of nonconjugated polymers is less well understood. Here CdS photocatalysts are coated by non-conjugated polyelectrolyte layers, and the influence of the polymer on charge transfer over CdS is explored.

## Introduction

Photocatalysis offers us a promising avenue with the tenet of sustainable chemistry toward substantial solar energy conversion^1–3^. Engineering the surface of pristine photocatalysts is a viable option to improve their photoactivities^4–6^. In recent years, polymers have been extensively investigated for photocatalytic applications by virtue of their enormous structural diversity and tunability in terms of potential properties or processability^7–9^. As a consequence of the delocalized *π* systems, conjugated polymers possess a one-dimensional (1D) band-like electronic structure that favors charge migration along the conjugated macromolecular backbone, making them suitable conductive charge mediators for charge separation/migration^10^. Nevertheless, such conjugated polymers suffer from prohibitive cost and scarcity, constraining the widespread deployment of polymer-based photosystems. It is noteworthy that recent studies about the surface chemical functional group modification of photocatalysts and electrocatalysts have pointed that the kinetics of interfacial charge transfers over catalysts are significantly influenced by the surface functional groups^11–14^. In these cases, surface functional groups with the charge-withdrawing capability provide “charge sink” platforms for accepting and transporting charges. For instance, Sun and colleagues^12^ reported that electron-donating groups attached to the backbone of the covalent-organic framework facilitate charge transfer inside the material^12^. Encouraged by this, we hope to investigate whether nonconjugated insulating polymers containing suitable functional groups (e.g., positively charged nitrogen moieties and amine groups) could induce molecular charge transfer as well as the conjugated polymers. In comparison with the conjugated polymers, the nonconjugated polyelectrolyte (NCP) interlayer has been widely used in recent years as the interface layer of various optoelectronic devices, such as organic solar cells^15^, organic light-emitting diodes^16^, and photoelectrocatalytic cells^17^, due to its low cost and excellent surface modification effects. Moreover, the polymer can be deposited onto the semiconductor surface via solution-processing methods such as spin-coating and drop-casting^18,19^, or vapor-based technologies such as evaporation^20,21^, or self-assembling strategies such as layer-by-layer assembly^22,23^, which do not require expensive apparatus and harsh experimental conditions, facilitating mass production. By attaching solid-state NCPs with charge-withdrawing pendant groups to the surface of semiconductor-based photocatalysts, the charge transfer between the semiconductor and pendant groups would take place while reducing the recombination rate of photoinduced charge carriers.

Yet, the role of NCPs as the charge transport mediator in photocatalysis, which is comparable to that of the conjugated polymers, has rarely been reported. An overview of previous works suggests that although some endeavors have been devoted to rationally designing the nonconjugated macromolecule-derived photocatalytic system with substantial solar energy conversion^24–28^, such as fabrication of multilayer photocatalysts by consecutive adsorption of nonconjugated polyanions and polycations^17,29^, an in-depth insight of charge-transfer kinetics at the molecular level in such photosystems is still elusive. As a result, it is important yet still challenging to unlock the relationship between molecular structure and charge transport characteristics, in order to identify suitable NCPs that might function as charge mediators in the photosystems.

In this contribution, rather than utilizing conjugated polymers, we modulate charge transfer over diverse semiconductors (i.e., CdS, TiO_2_, and Bi_2_WO_6_) by functionalizing the semiconductor surface with certain NCPs via facile ligand-triggered self-assembly strategy, wherein such NCPs consist of the nonconjugated backbone with charge-transporting sites. We found that the surface functionalization of semiconductors with polyethyleneimine (PEI) could lead to efficient hole transfer from semiconductors to solid-state insulating PEI layer, owing to the electron-donating capability of electron-rich amine groups from PEI, although solid-state nonconductive poly(allylamine hydrochloride) (PAH) layer could directionally extract electron from the semiconductor, most probably originating from the electron-withdrawing capability of the charged ammonium groups along the PAH backbone. Consequently, the resultant PEI-/PAH-modified nanocomposites exhibit superior charge separation efficiency, demonstrating improved photoredox performance. Up to now, to our knowledge, no attempts have been made to construct the nonconjugated insulating NCP-functionalized photocatalytic system for accelerating the interfacial charge transfer, although several nonconjugated polyanions and polycations have been reported to modify photocatalysts for organic dye degradation or H_2_O splitting without detailed mechanistic studies^27,30–32^.

## Results and discussion

### Preparation and structural characterizations of CdS@NCPs

**Fig. 1:**
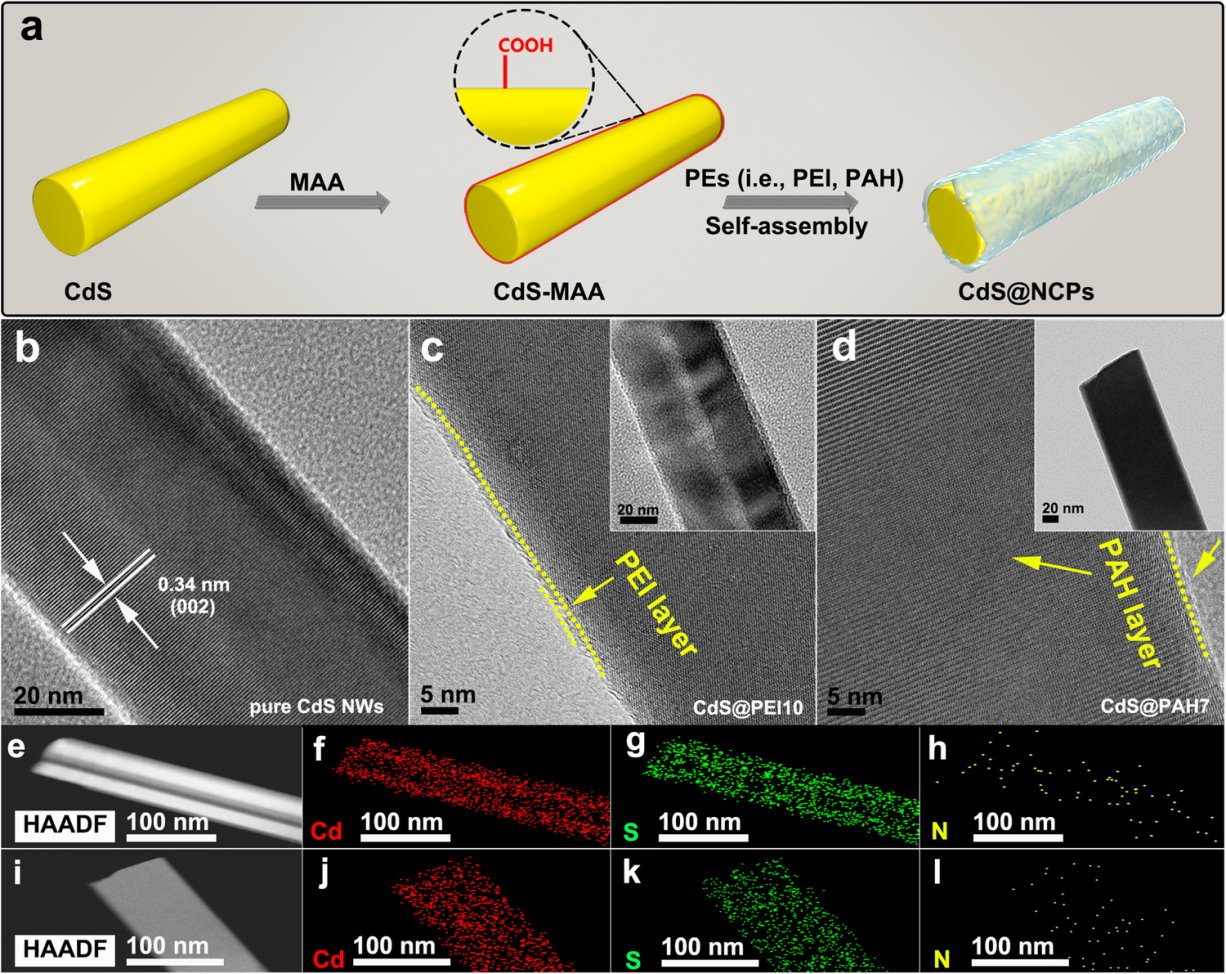
Synthesis and morphology characterization. **a** Schematic illustration of the formation process of PEI-/PAH-functionalized CdS NW composite catalysts. HRTEM images of **b** CdS NWs, **c** CdS@PEI10, and **d** CdS@PAH7. Insets in **c** and **d** show the TEM images of CdS@PEI10 and CdS@PAH7, respectively. Elemental mapping results of **e**–**h** CdS@PEI10 and **i**–**l** CdS@PAH7.

A ligand-triggered self-assembly strategy was developed to prepare the NCPs (i.e., PEI and PAH)-functionalized CdS nanowire (NW), as illustrated in Fig. 1a. To begin with, the as-synthesized CdS NW substrate was modified by a coupling agent, mercaptoacetic acid (MAA), to produce a surface with plentiful carboxyl groups exposed,which fosters the chemical attraction of the nitrogen moieties along with PEI or PAH backbone (Supplementary Figs. 1–3). Thereafter, the MAA-modified CdS NW was dispersed in a sodium chloride solution of NCPs, followed by thorough rinsing with ethanol. This removed NCP nonspecifically attached to the sample surface, leaving ultrathin layers of NCP, ranging down to several layers, irreversibly encapsulated on CdS NW. Due to the bonding force between the assembly units, the MAA-modified CdS NW was wrapped with a layer of the NCP, which results in the NCP-functionalized CdS NW nanocomposite, denoted as CdS@NCP. By adjusting the concentration of PEI and PAH aqueous solutions, a series of CdS@NCPs were achieved, respectively labeled as CdS@PEI*x* (*x* = 5, 10, and 15 mg mL^−1^) and CdS@PAH*y* (*y* = 5, 7, and 10 mg mL^−1^). Similarly, CdS NW substrate can be replaced by other semiconductor photocatalyst substrates (e.g., TiO_2_ and Bi_2_WO_6_) to construct diverse photocatalysts with surfaces functionalized by NCP via a similar self-assembly strategy. It is noteworthy that such a preparation method only involves adsorption from solution under ambient conditions without relatively complex synthetic procedures and prohibitive cost, featuring a facile and green process.

The transmission electron microscopy (TEM) images of bulk CdS NWs in Fig. 1b and Supplementary Fig. 4 present a typical 1D structure with a length of several hundred nanometers. From high-resolution TEM (HRTEM), such fine morphology featured by a rather smooth surface was observed from CdS NWs. The lattice fringe of 0.34 nm spacing is indexed to the (002) crystallographic plane of CdS (JCPDS number 41-1049, Supplementary Fig. 5)^33^. When NCP was deposited on the CdS NW substrate enabled by self-assembly, as seen in Supplementary Figs. 6 and 7, the morphologies of CdS@NCPs (i.e., CdS@PEI10 and CdS@PAH7) are analogous to that of CdS NWs due to the ultrathin thickness of the NCP layer. Fig. 1c, d show the HRTEM images of CdS@PEI10 and CdS@PAH7. In sharp contrast to the smooth surface and clear lattice fringe for the bulk CdS NWs, a rough sheath consisting of ultrathin amorphous NCP layer was formed on the CdS NW border for CdS@PEI10 and CdS@PAH7 because of the chemical interaction between the surface of CdS NW and the NCP layer. Simultaneously, the lattice spacings in CdS@PEI10 and CdS@PAH7 both become fuzzy as a result of the amorphous NCP shield. The encapsulation of a single CdS NW with NCP layer can also be validated by TEM elemental mapping images. Fig. 1e–l display the homogeneous distribution of Cd, S, and N elements on CdS@PEI10 and CdS@PAH7, respectively, among which the N signal results from the NCP layer, strongly substantiating the attachment of the NCP layer to the surface of CdS NW.

**Fig. 2:**
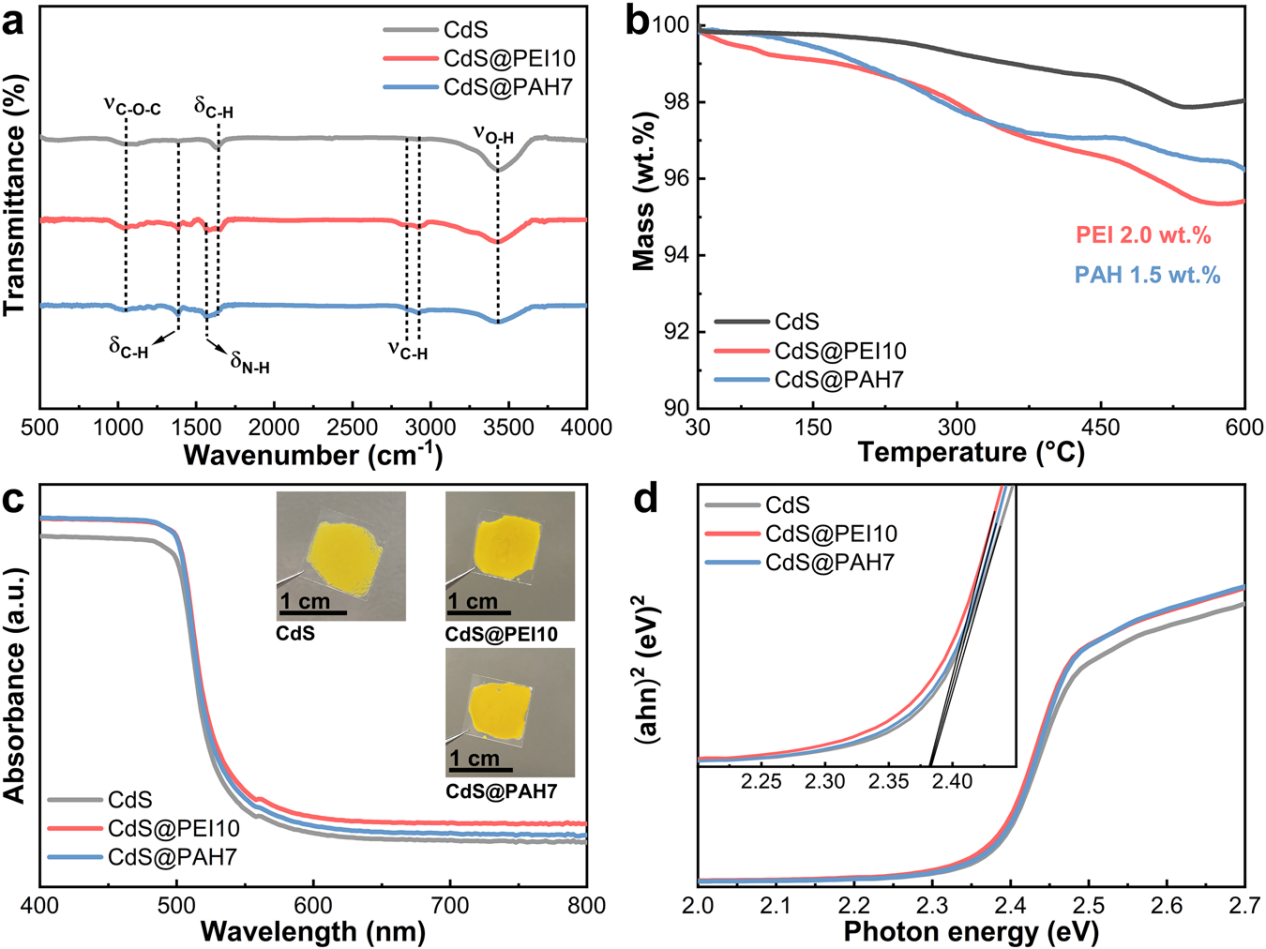
Spectroscopy characterization. **a** FTIR spectra, **b** TGA curves, **c** DRS results, and **d** bandgap determination of CdS NWs, CdS@PEI10, and CdS@PAH7.

The NCP-functionalized CdS NWs were further followed by Fourier transform infrared (FTIR) spectroscopic measurement. As displayed in Fig. 2a and Supplementary Table 1, the FTIR result of CdS NWs shows the peaks at 3436, 1631, and 1037 cm^−1^, which are assigned to the characteristic vibration modes of -OH and C-O-C, respectively^34^. As compared to the FTIR result of CdS NWs, the peaks at 2929, 2854, and 1384 cm^−1^ attributable to -CH_2_ groups from the molecular chains of PEI and PAH are visualized in the FTIR spectra of both CdS@PEI10 and CdS@PAH7^35,36^. Moreover, enriched characteristic vibration modes of PEI and PAH (e.g., N-H at 1584 cm^−1^) were also detected in the FTIR spectrum of the CdS@PEI10 and CdS@PAH7^37,38^. Thermogravimetric analyses for CdS@NCPs were conducted under nitrogen, to assess the mass ratio of NCP to CdS NWs, and the results are summarized in Fig. 2b. The mass loss below 100 °C could be attributed to the removal of adsorbed water. It is noted that both CdS@PEI10 and CdS@PAH7 show a two-step thermal degradation process with about 2.0 and 1.5 wt.% loss of PEI and PAH chains up to around 500 °C, respectively. The ultraviolet-visible (UV-vis) diffuse reflectance spectra results in Fig. 2c manifest that all photocatalysts show a significant light absorption in the wavelength region below ca. 520 nm, without discernible variation in the absorption band edge among CdS NWs, CdS@PEI10, and CdS@PAH7. This result is justified by the fact that PEI and PAH are both characterized by an amorphous polymer without light response within the visible spectral domain (Supplementary Fig. 8), and thus the absence of photoexcitation. Moreover, due to the ultrathin NCP layer, the nanocomposites retain good optical properties and approximately the same optical bandgap as the pure CdS NWs, as reflected by the Tauc plots in Fig. 2d. CdS@PEI10 and CdS@PAH7 exhibit higher absorption intensity compared with the pristine CdS NWs; this stems from the deepened color of CdS NWs after NCP layer encapsulation (insets in Fig. 2c).

### Electronic coupling interaction between NCPs and CdS

**Fig. 3:**
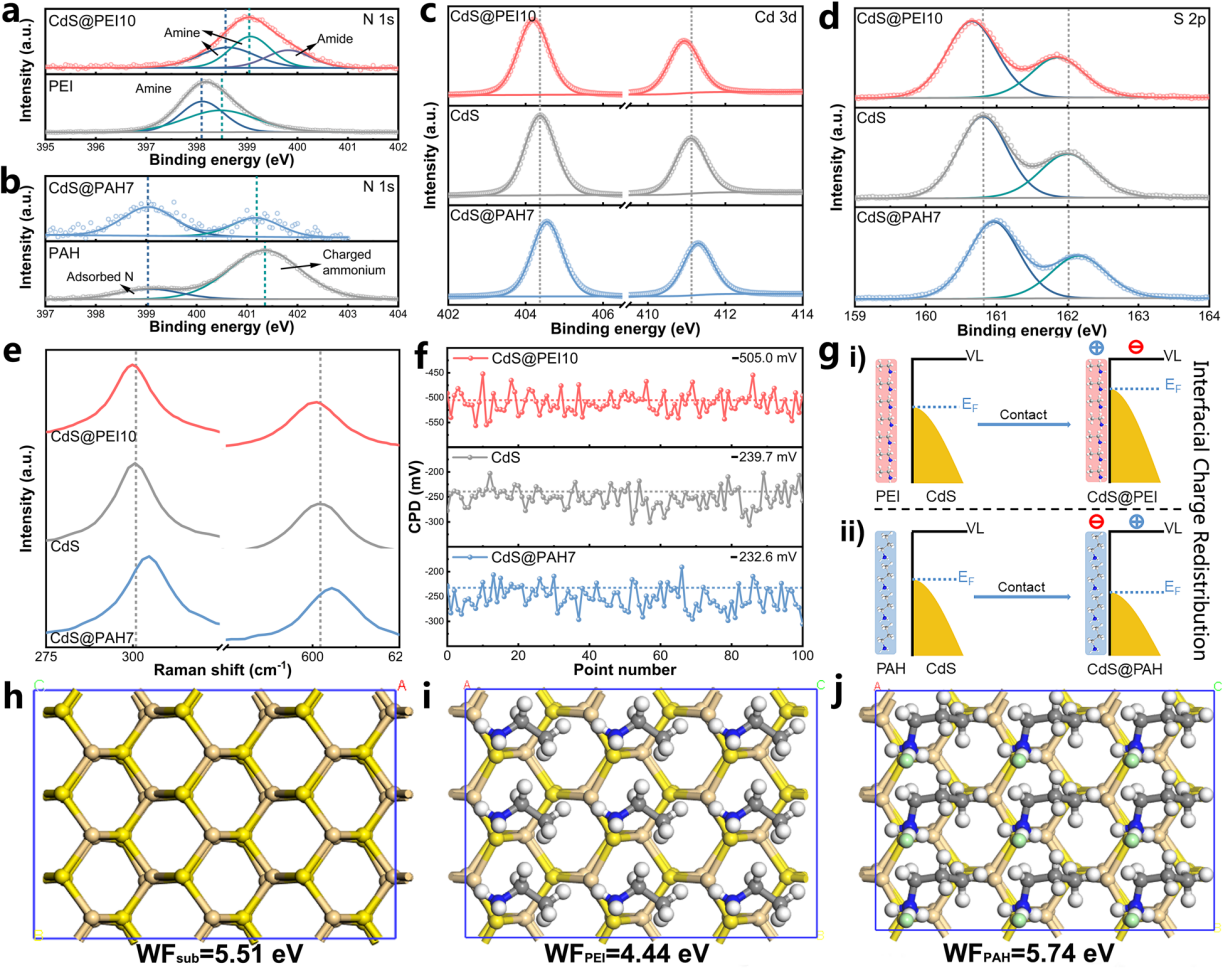
Charge transport characteristics of PEI and PAH. High-resolution N 1*s* spectra of **a** pure PEI and CdS@PEI10, and **b** pure PAH and CdS@PAH7. High-resolution **c** Cd 3*d* and **d** S 2*p* of CdS NWs, CdS@PEI10, and CdS@PAH7. **e** Raman spectra and **f** SKP measurement results of CdS NWs, CdS@PEI10, and CdS@PAH7. **g** The corresponding surface charge-transfer models of CdS@PEI10 and CdS@PAH7. The most stable structures of **h** pristine CdS (110), **i** PEI, and **j** PAH on the surface of CdS (110) with the corresponding calculated WF.

X-ray photoelectron spectroscopic (XPS) analysis was used to probe the interaction of CdS NWs and NCP layer. Survey XPS spectra of CdS NWs and CdS@NCPs demonstrate the Cd 3*d*, S 2*p*, and C 1*s* elements (Supplementary Fig. 9). Figure 3a shows the high-resolution N 1*s* XPS spectra of pure PEI and CdS@PEI10, in which the peaks at around 398.1 and 398.5 eV for pure PEI are attributed to the amine groups (-NH-/-NH_2_). As compared to the N 1*s* XPS peaks in pure PEI, the PEI N1*s* peak position for CdS@PEI10 shifts positively to the higher binding energy (B.E.) by ca. 0.5 eV and, simultaneously, the B.E. downshifts are observed in the high-resolution Cd 3*d* and S 2*p* spectra of the CdS@PEI10 with respect to those of the pristine CdS NWs (Fig. 3c, d and Supplementary Table 2). The increase of B.E. indicates the weakened electron screening effect due to the decrease of the electron concentration, whereas the decrease of B.E. means an increase of electron concentration^39^. This indicates the occurrence of hole transfer from CdS NWs to the N species of PEI, leading to the construction of a directional hole-transfer channel in CdS@PEI10, which could be explained by the electron-donating capability of electron-rich -NH-/-NH_2_ from PEI^40–44^. The electron donation resembles the case observed by a reducing agent adsorbed on CdS NWs that causes PEI to act as a hole reservoir for efficaciously capturing hole photoexcited from the CdS NW matrix. Moreover, the B.E. at 399.9 eV in high-resolution N 1*s* XPS spectra of CdS@PEI10 can be assigned to the amide group^45^, indicating that PEI was grafted on the CdS NW surface via the amide linkage owing to the chemical attraction between PEI and carboxyl groups on the surface of CdS NWs (Supplementary Fig. 2). With regard to the high-resolution N1*s* XPS spectrum of pure PAH, as displayed in Fig. 3b, the peak at 401.3 eV is attributable to the charged ammonium group (-NH_3_^+^)^42,46^, its negative shift of ca. 0.2 eV in CdS@PAH7 suggests the electron transfer from CdS NWs to the PAH, most probably arising from the strong electron-withdrawing capability of positively charged -NH_3_^+^ along the PAH backbone^41,42,47^. Besides, another peak at 399.0 eV is also observed in the high-resolution N 1*s* spectra of both PAH and CdS@PAH7, both of which are assigned to the adsorbed N species. As expected, the Cd 3*d* and S 2*p* peaks of CdS@PAH7 evolve toward the higher B.E. regions, suggesting that PAH has acted as an electron-transfer channel to cause the partial electron transfer from the CdS NW substrate. Such charge-transfer interactions were also verified by Raman spectroscopy. As displayed in Fig. 3e, the two distinct vibration peaks located at 301 and 602 cm^−1^ corresponding to the first order (1 LO) and second-order (2 LO) longitudinal optical phonon modes of CdS are observed in the Raman spectra of blank CdS NWs^48^. The two peaks, both show blueshifts after attaching the PEI to the CdS NW surface (1 and 2 cm^−1^ for 1 LO and 2 LO, respectively), whereas the redshifts are seen in the 1 LO and 2 LO peaks of the CdS NWs after PAH encapsulation (4 and 3 cm^−1^ for 1 LO and 2 LO, respectively), further substantiating the occurrence of a strong charge coupling interaction between the CdS NWs and PEI/PAH layer.

To validate the charge-transfer interaction that occurred at the CdS@NCPs interface, scanning Kelvin probe measurement was conducted to probe the variation in local work function (WF) of the CdS NW surface before and after NCPs coating. In general, the change in WF of the sample is related to the variation of the contact potential difference (CPD) between the sample surface and the conductive tip as a reference (*φ*_tip_ − *φ*_sample_)^49^. Figure 3f demonstrates the CPD results of CdS NWs and PEI-/PAH-functionalized CdS NWs composite catalysts collected from 100 different points, with calculated mean CPD values given in the legend. For pristine CdS NWs, the mean CPD value is calculated to be −239.7 mV. After encapsulation with PEI, the CPD value of CdS@PEI10 sharply decreases to −505.0 mV; this implies a lower WF in the PEI-functionalized CdS NWs, which is energetically favorable for the hole migration from CdS NWs to PEI layer. Oppositely, the CPD value shows an upshift to −232.6 mV after anchoring the PAH layer onto the CdS NWs surface, substantiating the presence of the electron-transfer interaction from CdS NWs to the PAH layer. This result once again proves that NCPs function as a charge acceptor (i.e., hole acceptor for PEI, electron acceptor for PAH) and attract charge from neighboring CdS NWs, building the hole accelerated PEI surface in CdS@PEI10 and electron accelerated PAH surface in CdS@PAH7 (Fig. 3g), which coincides with the above spectroscopic investigation and further density functional theory (DFT) calculations. As demonstrated in Fig. 3h–j, the variation trend of WF via DFT calculations is in high agreement with the CPD result. The most stable fractional coordinates for PEI/PAH adsorbates on CdS (110) surface see Supplementary Data 1.

### Interfacial charge separation efficiency of CdS@NCPs

**Fig. 4:**
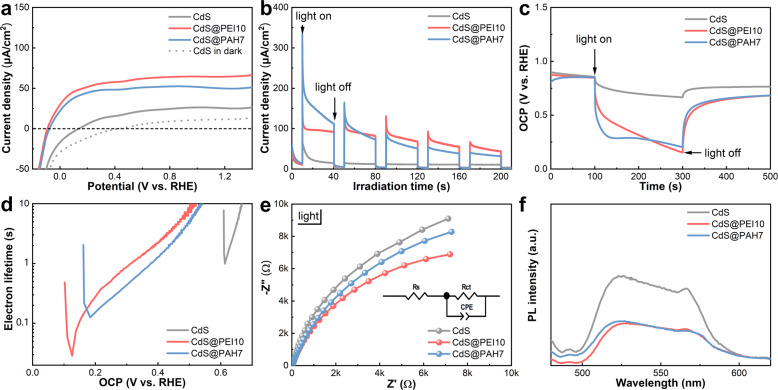
Charge recombination kinetic analysis. **a** LSV (scan rate: 5 mV s^−1^), **b** *I*–*t* (bias: 1.2 V vs. RHE), **c** OCPD, **d** electron lifetime, and **e** EIS Nyquist plots (bias: 0.6 V vs. RHE) of CdS NWs, CdS@PEI10, and CdS@PAH7 photoanodes under visible-light irradiation in 0.5 M Na_2_SO_4_ solution. **f** PL emission spectra of the same three samples with an excitation wavelength of 350 nm. The insets in **e** demonstrate the equivalent circuit of the above photoanodes.

Photoelectrochemical (PEC) results were collected to evaluate the interfacial charge separation efficiency of CdS@NCPs nanocomposite. We first tested the photocurrent of the photoanodes by linear sweep photovoltammetry (LSV) and on–off transient photocurrent response (*I*–*t*) measurements. As can be seen from the LSV curves under visible-light irradiation displayed in Fig. 4a, CdS@PEI10 and CdS@PAH7 yield substantially enhanced photocurrent with early-onset potential in comparison with the pristine CdS NWs under the same conditions, whereas only weak photocurrents are observed in the dark (Supplementary Fig. 10). The improved photocurrent and lower-onset potential imply more effective charge separation efficiency over CdS@PEI10 and CdS@PAH7 than CdS NWs, manifesting that the functionalization of CdS NWs with PEI or PAH can suppress the charge combination by promoting the interfacial charge transfer between CdS NWs and NCP layer. Figure 4b demonstrates the photocurrent of different photoanodes at 1.2 *V*_RHE_ under chopped visible-light irradiation, wherein CdS@PEI10 and CdS@PAH7 also show remarkably improved photocurrents as compared with the blank CdS NWs. It is noteworthy that the negligible photocurrent of pure PEI and PAH photoanodes means that the synergistic interaction of the CdS NW matrix and ultrathin NCP layer, in particular the interfacial charge transfer between them, is required for the considerably increased PEC activity of CdS@PEI10 and CdS@PAH7 (Supplementary Fig. 11).

To reveal the charge-transfer kinetics, light-induced open-circuit photovoltage decay for the photocatalysts was conducted, which can be harnessed to monitor the charge decay process and evaluate the electron lifetime^50^. The decay rate of photovoltage is associated with the electron lifetime and their correlation can be expressed by the equation below^51^:

$$\tau = \frac{{{{{{k}}}}_{{{{{{{\mathrm{B}}}}}}}}T}}{{{{{e}}}}}\left( {\frac{{{{{{{{{\mathrm{d}}}}}}}}V_{{{{{{{{\mathrm{oc}}}}}}}}}}}{{{{{{{{{\mathrm{d}}}}}}}}t}}} \right)^{ - 1}$$

where *τ* is the electron lifetime, *k*_B_*T* is the thermal energy, *e* is the electron charge, and *V*_oc_ is the open-circuit photovoltage. As portrayed in Fig. 4c, CdS@PEI10 and CdS@PAH7 exhibit higher photovoltage, slower photovoltage decay rate, and longer electron lifetime (Fig. 4d) than blank CdS NWs under visible-light irradiation, corroborating their more enhanced charge separation. Furthermore, the crucial roles of the solid-state nonconductive ultrathin PEI and PAH layers as the charge-transfer mediator can be verified by the electrochemical impedance spectroscopy (EIS) results. As displayed in Supplementary Fig. 12, the semicircular arc radius of the EIS curves for the blank CdS NWs, CdS@PEI10, and CdS@PAH7 electrodes was probed in the dark, which shows that CdS@PEI10 and CdS@PAH7 have larger semicircle arc radius than blank CdS NWs. Apparently, PEI and PAH layers as the solid-state insulating polymer covered on the CdS NW substrate surface increase the interfacial charge-transfer resistance of the electrodes (Supplementary Table 3). However, intriguingly, the charge-transfer resistances of CdS@PEI10 and CdS@PAH7 are considerably decreased relative to that of blank CdS NWs upon light irradiation (Fig. 4e and Supplementary Table 4), suggesting the existence of interfacial charge transfer between the solid-state NCP layer and the semiconductor due to the molecular interaction, in which PEI and PAH serve as the charge-transfer channel, resulting in significantly enhanced interfacial charge-transfer/separation efficiency. Alternatively, as revealed in Fig. 4f, CdS@PEI10 and CdS@PAH7 yield lower photoluminescence (PL) intensity than that of pure CdS under identical conditions; a decrease in radiative recombination rate seems to be the reason for the decreased PL intensity, which is likely linked to the separation of charge carriers that otherwise could recombine radiatively.

### Photocatalytic performances and mechanism of CdS@NCPs

**Fig. 5:**
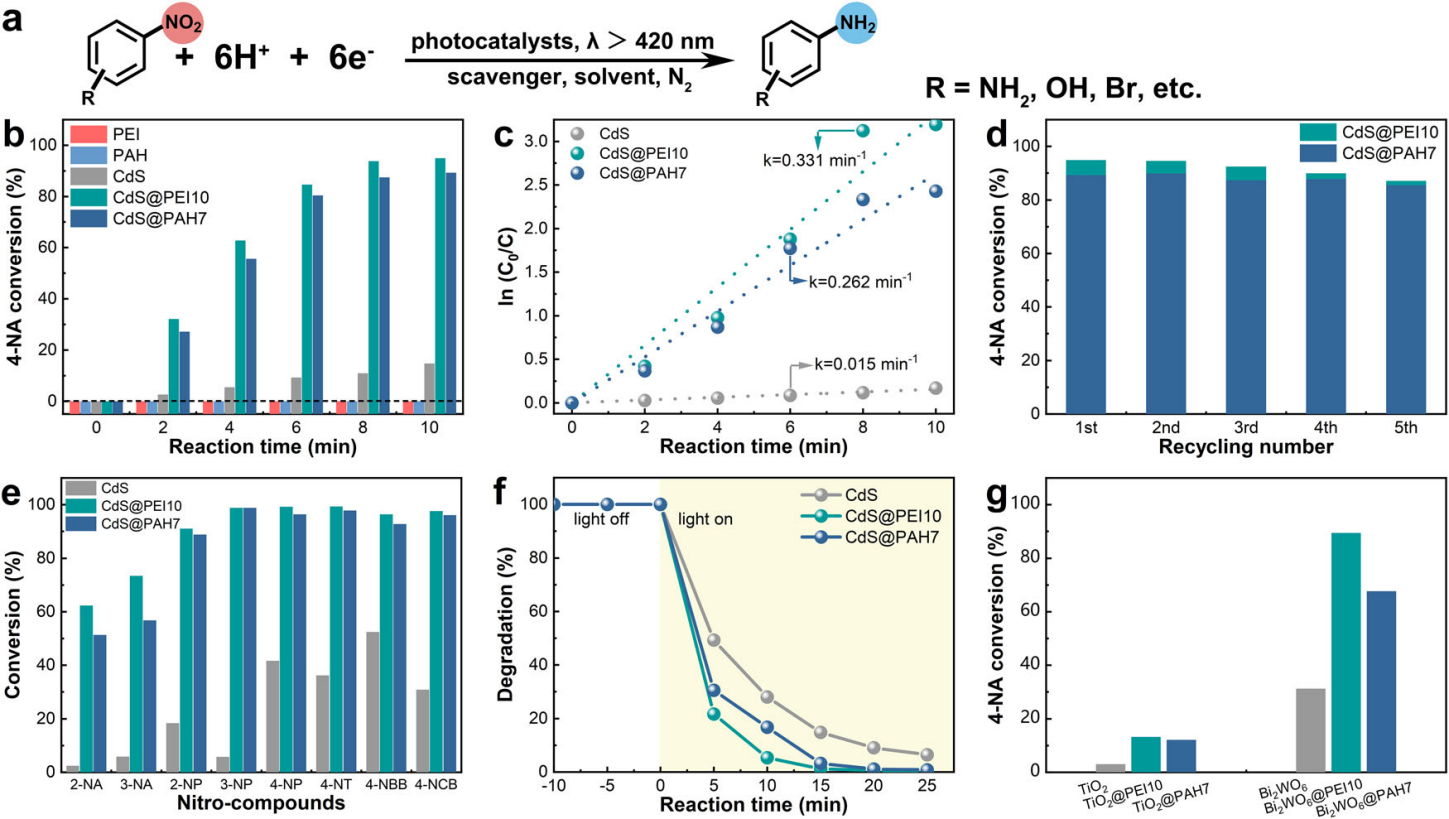
Photocatalytic activity. **a** Photoreduction reaction model of 4-NA. **b** Photoactivities of blank CdS NWs, CdS@PEI10, and CdS@PAH7 toward selective reduction of 4-NA together with the corresponding **c** kinetic curves. **d** Cyclic photoreduction performances of CdS@PEI10 and CdS@PAH7. Photoactivities of the same three samples toward **e** selective reduction of aromatic nitro compounds and **f** mineralization of RhB. **g** Photoactivities of other PEI-/PAH-functionalized semiconductors composite catalysts toward photoreduction of 4-NA.

The photoreduction of 4-nitroaniline (4-NA) to 4-phenylenediamine performance of PEI-/PAH-functionalized CdS NWs was initially evaluated to unlock the synergistic interaction between PEI/PAH and CdS NWs in influencing the photoactivities of the composite catalysts (Fig. 5a). The photoreduction performances can be monitored by UV-vis absorption spectroscopy (Supplementary Fig. 13). Blank experiments in the absence of a catalyst or light (Supplementary Fig. 14) corroborate that the reaction is a photocatalytic process. As displayed in Supplementary Figs. 15 and 16, and in Supplementary Table 5, the photoactivities of CdS@NCPs in terms of conversion have a close relationship with the encapsulation concentration of NCPs, wherein CdS@PEI10 and CdS@PAH7 exhibit remarkably enhanced photoactivities with excellent reaction rate in contrast with the unitary CdS NWs under the same experimental conditions. The photoactivities of PEI, PAH, and PEI10-/PAH7-functionalized CdS NWs are summarized in Fig. 5b with the corresponding kinetic curves in Fig. 5c. Our catalyst outperforms many previously reported CdS-based photocatalysts (Supplementary Table 6). It is well known that an efficient charge separation/transfer is crucial for the enhancement of photocatalytic performance. Considering that PEI or PAH alone shows no photoactivity and pure CdS NWs demonstrate inferior conversion efficiency of 4-NA, it is undoubtedly revealed that the substantially boosted photoactivities of CdS@PEI10 and CdS@PAH7 are due primarily to the boosted charge separation. The stability of the composite catalysts was also tested. As revealed from Fig. 5d, CdS@PEI10 and CdS@PAH7 demonstrate relatively favorable photostability with a slight photoactivity decay after five successive cyclic reactions. The FTIR results of CdS@PEI10 and CdS@PAH7 after cyclic photoreaction were analyzed. As revealed in Supplementary Fig. 17, the peak intensity and position of typical functional groups show slight changes after cyclic reaction, which could be attributed to the loss of PEI/PAH layer or degradation of PEI/PAH layer during the cyclic reaction. Nonetheless, it should be noted that no apparent new peaks were observed in the FTIR spectra. Moreover, the XRD patterns of fresh and used CdS@NCPs demonstrate an identical crystalline structure, and the elemental chemical states of the PEI/PAH layer hardly changed before and after cyclic photoreaction (Supplementary Figs. 18 and 19), verifying the relatively favorable photostability of the catalyst. Alternatively, as shown in Fig. 5e and Supplementary Fig. 20, apart from 4-NA, PEI-/PAH-functionalized CdS NWs also exhibit considerably enhanced photoactivities toward reduction of other nitroaromatics in comparison with pristine CdS NWs. More significantly enhanced photocatalytic performances of CdS@PEI10 and CdS@PAH7 relative to blank CdS NWs were also observed in the mineralization of RhB under visible-light irradiation, which manifests the general role of PEI/PAH in boosting the interfacial charge separation/transfer (Fig. 5f and Supplementary Fig. 21).

The charge transfer between CdS NW and NCPs layer can further be experimentally proven by the 4-NA photoreduction performance of composite catalysts prepared by incorporating an ultrathin silica layer into the interface of CdS and NCPs. As revealed in Supplementary Fig. 22, the reference samples CdS@SiO_2_@NCPs (i.e., CdS@SiO_2_@PEI10 and CdS@SiO_2_@PAH7) exhibit minimal photoactivity under the same conditions, which is due to the steric effect induced by the SiO_2_ insulation shell, thereby conspicuously blocking the charge-transfer channel between CdS NW and NCP layer, significantly lowering the photoactivity. It is worth noting that such charge-transfer interaction did not seem to be due to the gradient energy level between CdS NWs and PEI/PAH layer, as neither PEI nor PAH possesses the delocalized *π* systems. Recently, Yu and colleagues^41^ have discussed the possible charge-transfer mechanism between carbon nanotubes and poly(diallyldimethylammonium chloride) (PDDA), suggesting that the charge-withdrawing ability of functional groups along the PDDA backbone led to the efficient electronic coupling interaction between carbon nanotubes and PDDA. Hence, the charge-transfer mechanism, arising from the charge-transporting functional groups of the nonconjugated macromolecules, is proposed to explain this result. To be specific, the amine group in PEI can donate its lone pair electrons to CdS NWs or accept hole from CdS NWs. In PAH, the lone pairs are occupied by H^+^ and thus the ammonium cations in PAH are positively charged, which is anticipated to withdraw electrons from the CdS NWs, thereby remarkably retarding the recombination of photoinduced charge carriers.

Taken together, it is beyond doubt that the ultrathin PEI and PAH interlayer can act as efficient “charge sink” platforms to boost the separation efficiency of photoinduced electron-hole pair, leading to gratifyingly enhanced photocatalytic performance. Furthermore, other classical photocatalysts (i.e., TiO_2_ and Bi_2_WO_6_) were also used as substrates for similar surface functionalization of semiconductor@NCPs (i.e., TiO_2_@PEI10, TiO_2_@PAH7, Bi_2_WO_6_@PEI10, and Bi_2_WO_6_@PAH7) composite catalysts, in order to validate the universality of the surface functionalization strategy. As shown in Fig. 5g, similar photoactivity enhancement was observed in other semiconductor@NCPs for 4-NA photocatalysis, verifying that such insulating NCP composed of the nonconjugated backbone with charge-transporting sites is able to induce interfacial charge transfer and can be used to construct a wide range of nonconjugated insulated NCP-functionalized photocatalytic systems for accelerating the interfacial charge transfer.

## Conclusions

In conclusion, we have adopted a certain class of nonconjugated macromolecules with charge-transporting functional groups to exquisitely modulate charge transfer over several semiconductors (i.e., CdS, TiO_2_, and Bi_2_WO_6_) via functionalizing the surface by a facile ligand-triggered self-assembly strategy, wherein such NCPs (i.e., PEI and PAH) can act as an efficient “charge sink” platforms for accelerating the interfacial charge transfer and thus retarding the recombination of photoinduced charge carriers over composite catalysts. As revealed by our experimental studies, due to the electron-donating capability of electron-rich amine groups from the PEI macromolecular backbone, surface functionalization of these semiconductors with PEI could lead to efficient hole transfer from semiconductor to solid-state insulating PEI layer, whereas solid-state nonconductive PAH layer could directionally extract electron from semiconductor, owing to the strong electron-withdrawing capability of charged ammonium groups along the PAH backbone. As a result, the optimal PEI-/PAH-functionalized semiconductor exhibits conspicuously enhanced versatile visible-light-driven photoredox performance. Moreover, we show that this facile, eco-friendly, and scalable approach is universal for designing various polymer-incorporated photosystems. Our work would shed light on the potential application of such solid-state insulating nonconjugated macromolecule as a novel conductive charge mediator in the photosystem for solar energy conversion.

## Methods

### Syntheses

PEI-/PAH-functionalized CdS NWs were prepared by a simple ligand-triggered self-assembly strategy at ambient conditions. Specifically, as-synthesized CdS NW (100 mg) substrate (the information on the fabrication of CdS NW is provided in the Supplementary Methods) was dispersed in deionized water (100 mL) by sonication for 30 min and then MAA aqueous solution (9 mL, 1 mol L^−1^) was added under vigorous stirring at room temperature. After stirring for 2 h, the MAA-modified CdS NWs were sufficiently rinsed with ethanol to wash away any redundant MAA moiety and finally dried at 333 K in an oven. CdS@NCPs with different concentrations of the NCPs were fabricated by the self-assembly method. Typically, a predetermined amount of NCP (PEI or PAH) was dissolved in sodium chloride solution (0.5 M, 50 mL) by stirring for 10 min and then MAA-modified CdS NWs (100 mg) was added under stirring for 2 h. Finally, the mixture was centrifuged and dried in an oven at 333 K. By varying the concentration of the NCPs solution, a series of photocatalysts were obtained. Alternatively, preparations of other semiconductors capped with NCPs are similar to that of CdS@NCPs.

### PEC measurements

All PEC measurements were performed on a CHI760D (Instruments, Inc., Shanghai, China) electrochemical workstation with a standard three-electrode system. The photocatalyst was used as the working electrode, whereas a platinum sheet (1.0 cm × 1.0 cm) and Ag/AgCl electrode served as the counter electrode and the reference electrode, respectively. The working electrode was prepared by dropping the photocatalysts (1.0 cm × 1.0 cm) slurry on Indium tin oxide (ITO) substrate (the information on the fabrication of working electrode is provided in the Supplementary Methods). Na_2_SO_4_ was used as the electrolyte (0.5 M). EIS Nyquist plots of the photoelectrodes were measured at 0.6 V vs. reversible hydrogen electrode (RHE). The amplitude of the sinusoidal wave was set at 5 mV and the frequency was varied from 1 × 10^6^ to 0.1 Hz.

### Photocatalytic redox reactions

The photoreduction activity of the photocatalysts was investigated by the photoreduction of 4-NA under visible-light irradiation (300 W xenon lamp coupled with a 420 nm cutoff filter). Typically, the sample (10 mg) and ammonium formate (40 mg) were added into 4-NA aqueous solution (30 mL, 20 p.p.m.). Prior to photoirradiation, the suspension was magnetically agitated in the dark for 60 min to establish the adsorption–desorption equilibrium between reactants and catalysts. At varied irradiation time intervals (0, 2, 4, 6, 8, and 10 min), an aliquot of the mixed solution (3 mL) was collected and centrifuged, and then monitored on a UV-Vis spectrophotometer. The entire experiment was carried out with N_2_ bubbling at a rate of 80 mL min^−1^.

The photooxidation activity of the photocatalysts was investigated by the photocatalytic mineralization of RhB at ambient conditions under visible-light irradiation (300 W xenon lamp coupled with a 420 nm cutoff filter). Typically, the sample (20 mg) was distributed in RhB aqueous solution (40 mL, 5 p.p.m.). Prior to photoirradiation, the suspension was magnetically agitated in the dark for 30 min h to establish the adsorption–desorption equilibrium between reactants and catalysts. At varied irradiation time intervals (0, 5, 10, 15, 20, and 25 min), an aliquot of the mixed solution (3 mL) was removed and centrifuged. A UV-Vis spectrophotometer was used to measure the change of the remaining RhB solution’s characteristic light absorption at 555 nm.

## Supplementary information

Supplementary Information

Description of Additional Supplementary Files

Supplementary Data 1

## Data Availability

The authors declare that all the data supporting the findings of this study are available within the article or in the Supplementary Information and Supplementary Data 1. Extra data are available from the corresponding author upon reasonable request.
