# Supplementary material for: Accelerating charge transfer via nonconjugated polyelectrolyte interlayers toward efficient versatile photoredox catalysis
Source: Commun Chem. 2021 Oct 22;4:150. doi: 10.1038/s42004-021-00589-w (PMC9814354; doi:10.1038/s42004-021-00589-w)
Supplement: Supplementary file 1 — Supplementary Information [file 42004_2021_589_MOESM1_ESM.pdf]

# Supporting Information

# Accelerating Charge Transfer via Nonconjugated Polyelectrolyte interlayers toward Efficient Versatile Photoredox Catalysis

Tao Li[1], Chuang Feng[1], Boon Kar Yap[1,2,3], Xuhui Zhu[1], Biquan Xiong[4], Zhicai He[1]*, Wai-Yeung Wong[4]*

1. Institute of Polymer Optoelectronic Materials and Devices, State Key Laboratory of Luminescent Materials and Devices, School of Material Science and Engineering, South China University of Technology, Guangzhou 510640, P. R. China.

E-mail: zhicaihe@scut.edu.cn

2. Electronic and Communications Department, College of Engineering, Universiti Tenaga Nasional, Kajang, Selangor 43000, Malaysia.

3. Institute of Sustainable Energy, Universiti Tenaga Nasional, Kajang, Selangor 43000, Malaysia.

4. Department of Applied Biology and Chemical Technology and Research Institute for Smart Energy, The Hong Kong Polytechnic University, Hung Hom, Hong Kong, P. R. China.

E-mail: wai-yeung.wong@polyu.edu.hk

## Supplementary Methods

### Materials

Sodium diethyldithiocarbamate trihydrate ($C_5H_{10}NNaS_2·3H_2O$), cadmium chloride ($CdCl_2·2.5H_2O$), ethylenediamine ($C_2H_8N_2$), mercaptoacetic acid (MAA: $C_2H_4O_2S$), ammonium formate ($NH_4HCO_2$), and potassium persulfate ($K_2S_2O_8$) were obtained from Sinopharm Chemical Reagent Co., Ltd. 2-Nitroaniline (2-NA), 3-nitroaniline (3-NA), 4-nitroaniline (4-NA), 2-nitrophenol (2-NP), 3-nitrophenol (3-NP), 4-nitrophenol (4-NP), 4-nitrotoluene (4-NT), 4-nitrochlorobenzene (4-NCB), 4-nitrobromobenzene (4-NBB), and RhB were purchased from Aladdin. Polyethyleneimine (PEI) and poly(allylamine hydrochloride) (PAH) were purchased from Alfa Aesar Chemicals Co., Ltd. Deionized water (DI $H_2O$, 18.2 MΩ·cm resistivity, Millipore) was obtained from local sources.

### Syntheses

(I) *Fabrication of CdS NWs*. CdS NWs were prepared by a hydrothermal method.[1] Typically, 1.124 g of cadmium diethyldithiocarbamate [$Cd(S_2CNEt_2)_2$], prepared by precipitation from a stoichiometric mixture of sodium diethyldithiocarbamate trihydrate and cadmium chloride in DI $H_2O$, was added into a Teflon-lined stainless-steel autoclave with a capacity of 50 mL. Then, the autoclave was filled with 40 mL of ethylenediamine to 80% of the total volume. The autoclave was maintained at 453 K for 24 h and then allowed to cool to room temperature. A yellowish precipitate was collected, washed with absolute ethanol and DI $H_2O$ to remove residue of organic solvents. The final products were dried in an oven at 333 K for 12 h.

(II) *Fabrication of $TiO_2$ spheres*. $TiO_2$ spheres were synthesized according to a previous report.[2] The amorphous precursors of $TiO_2$ were prepared via a sol-gel strategy. Typically, 1.75 g hexadecylamine was first added and dissolved in 200 mL ethanol, followed by the injection of a potassium chloride solution (0.1 M, 0.8 mL). Then, 4.32 mL titanium isopropoxide was quickly injected into the solution along with vigorous stirring under an ambient temperature. The as-obtained suspension was kept static for 12 h and then washed with ethanol and dried in an oven at 60 °C. $TiO_2$ spheres were prepared through a solvothermal treatment of the

precursors and calcination. Typically, 1.6 g amorphous precursors were dispersed in a mixture of 20 mL ethanol and 10 mL DI water and then sealed within a 50 mL Teflon-lined autoclave and heated to 160 °C for 16 h. After washing and drying, the powders were calcined at 500 °C for 2 h in the air to remove organic components and form the $TiO_2$ products.

(III) *Fabrication of $Bi_2WO_6$ sheets*. The ultrathin $Bi_2WO_6$ sheets were synthesized via a simple hydrothermal method.[3] Typically, 83 mg of sodium tungstate dihydrate (0.25 mmol) was added into a 10 mL DI $H_2O$. Then, a solution of bismuth nitrate (0.05 M, 10 mL was added dropwise to the above solution. After stirring for 30 min at 40 °C, the mixed suspension was transferred into a 25 mL Teflon-lined autoclave and treated at 120 °C for 24 h. Finally, the products were collected, washed, and dried at 60 °C for 10 h.

**Characterizations**

Transmission electron microscopy (TEM), high-resolution (HR) TEM, and element mapping images were obtained using a JEOL-2010 instrument at an accelerating voltage of 200 kV. Crystal phases of the samples were determined by X-ray diffraction (XRD, X'Pert Pro MPD, Philips, Holland) using Cu Kα radiation at 40 kV and 40 mA. Thermogravimetric analysis (TGA) was carried out on a TG209F1 thermal analyzer at a heating rate of 10°C $min^{-1}$ in $N_2$. Fourier transform infrared (FTIR) spectroscopy measurements were performed on a TJ270-30A spectrophotometer at a resolution of 4 $cm^{-1}$. Raman spectra were measured on a Renishaw inVia spectrometer. The optical properties of the samples were probed by a Cary 500 UV-vis diffuse reflectance spectroscopy (DRS), in which $BaSO_4$ was employed as the internal reflectance standard. X-ray photoelectron spectroscopy (XPS) measurements were carried out on a Thermo Scientific ESCALAB250 spectrometer using Al Kα radiation source and binding energies (B.E.) of elements were calibrated by the B.E. of carbon at 284.6 eV. The contact potential difference (CPD) between the sample surface and the conductive tip was carried out using scanning Kelvin probe (SKP) microscopy from KP Technology, United Kingdom. Photoluminescence (PL) spectra were acquired on a Varian Cary Eclipse spectrometer with an excitation wavelength of 350 nm.

**Fabrication of working electrodes**

The working electrode was prepared on indium tin oxide (ITO) glass that was cleaned by sonication in ethanol for 30 min and dried at 353 K. The boundary of ITO glass was protected using scotch tape. The 5 mg sample was dispersed in 0.5 mL of ethyl alcohol absolute by sonication to get slurry. The slurry was spread onto the pretreated ITO glass. After air drying, the Scotch tape was unstuck, and the uncoated part of the electrode was isolated with nail polish. The exposed area of the working electrode was 1 $cm^2$. Finally, the working electrode was vertically dipped into the electrolyte and irradiated with visible light.

**Density function theory (DFT) calculations**

All the calculations were performed based on the simulation package of CASTEP.[4] The exchange and correlation potential was described by the generalized gradient approximation (GGA) with Perdew-Burke-Ernzerhof (PBE) method.[5] Moreover, the method of Grimme[6] was employed to describe the van der Waals force. The convergence tolerance of energy, max force, and max displacement was set to $1.0 \times 10^{-5}$ eV/Å, 0.03 eV/Å, and 0.001 Å, respectively. In order to avoid the interference of adjacent cells, a 25 Å vacuum layer was constructed. For the adsorption models of PEI and PAH, a monomer molecule was placed on the surface of CdS (110). The most stable structures of PEI and PAH on the surface of CdS (110) can be obtained by the structure optimization.

## Supplementary Discussion

The FTIR result of CdS NWs shows the peaks at 3436, 1631, and 1037 $cm^{-1}$, which assign to the characteristic vibration modes of -OH and C-O-C, respectively. Notably, no new peak was observed in the FTIR spectrum of the MAA-CdS with respect to that of the blank CdS NWs; this is primarily due to the low loading amount of MAA molecules on the surface of the CdS NWs. However, it should be stressed that the peak intensity at 1631 $cm^{-1}$ for the blank CdS NWs increased to some extent after MAA modification, which results from the contribution of the C=O stretching vibration mode (-COOH) from MAA molecules; this substantiates the adsorption of MAA molecules on the CdS NWs.

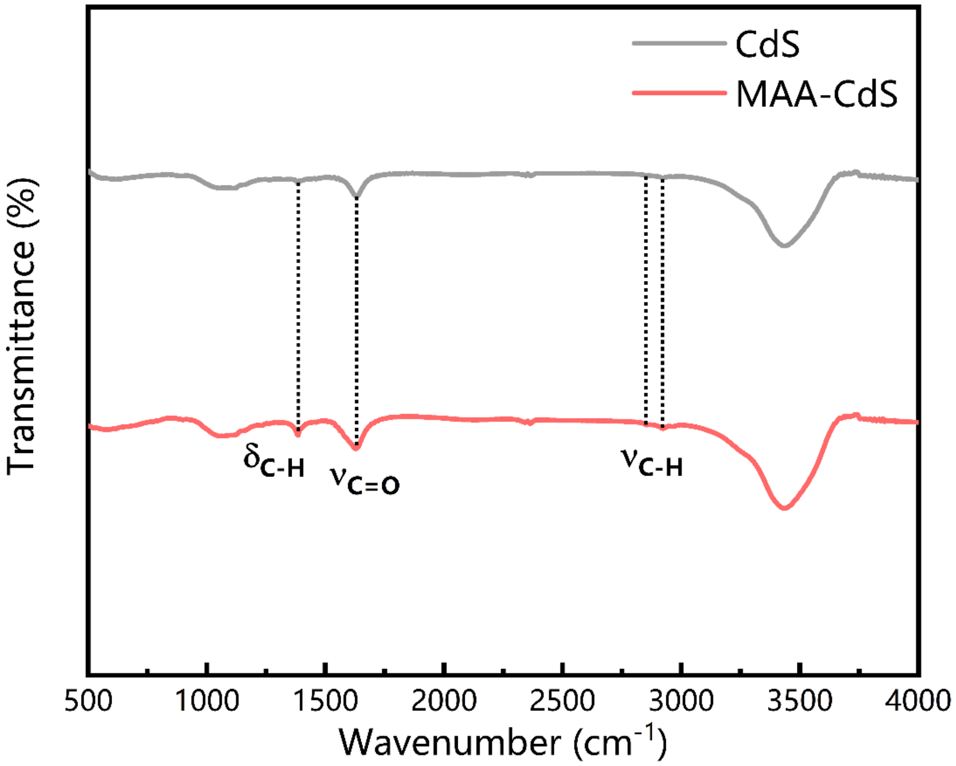


**Supplementary Fig. 1** FTIR spectra of CdS NWs and MAA-modified CdS NWs.

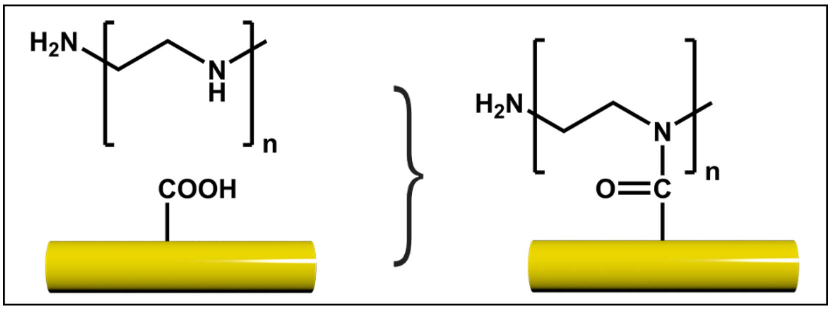


**Supplementary Fig. 2** Stacking mode of X@PEI (X: CdS, $TiO_2$, $BiWO_6$) composite catalysts.

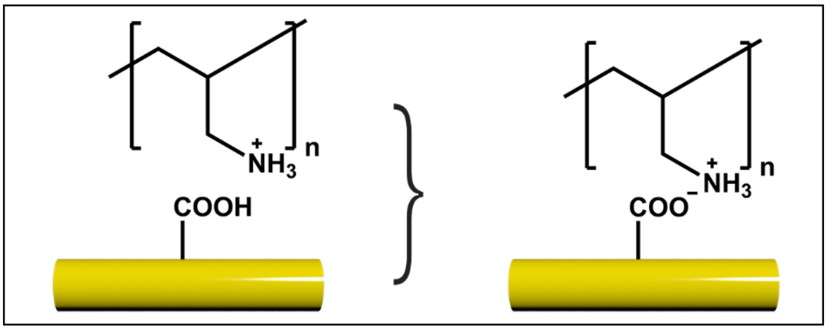


**Supplementary Fig. 3** Stacking mode of X@PAH (X: CdS, $TiO_2$, $BiWO_6$) composite catalysts.

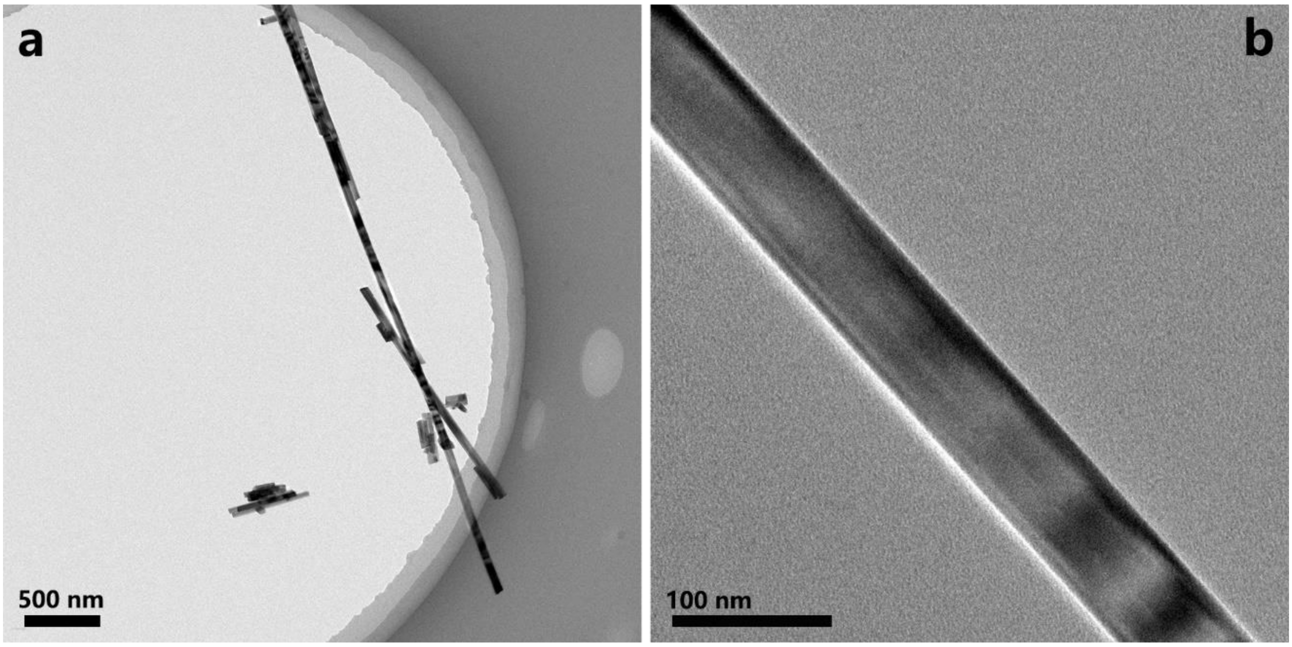


**Supplementary Fig. 4** TEM images of pure CdS NWs.

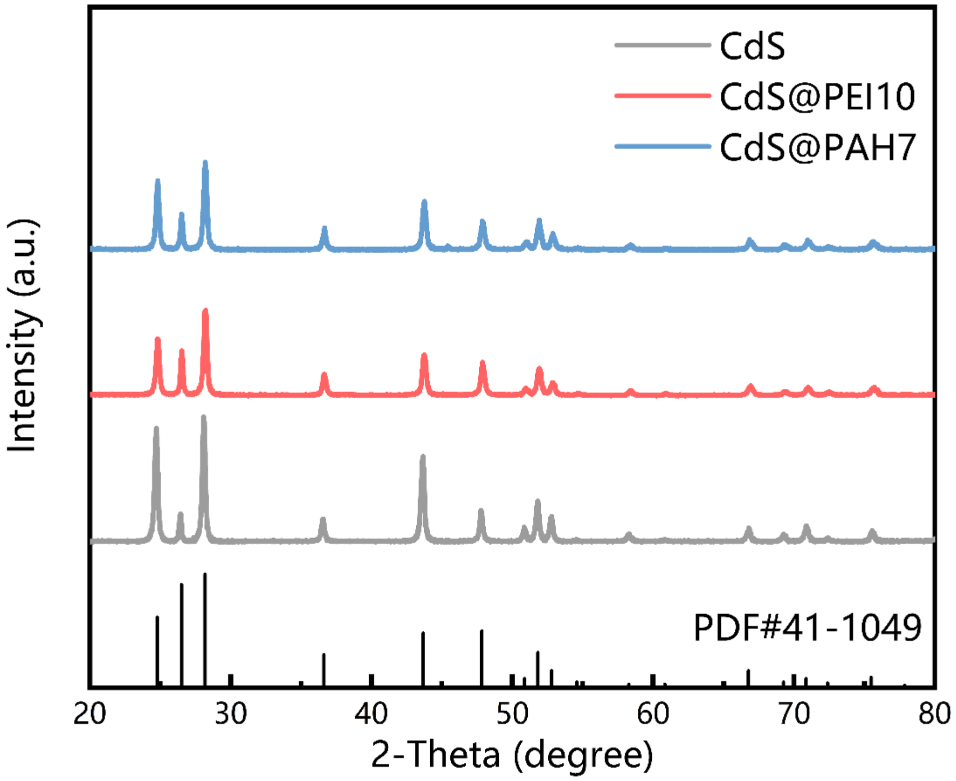


**Supplementary Fig. 5** XRD patterns of CdS NWs, CdS@PEI10, and CdS@PAH7.

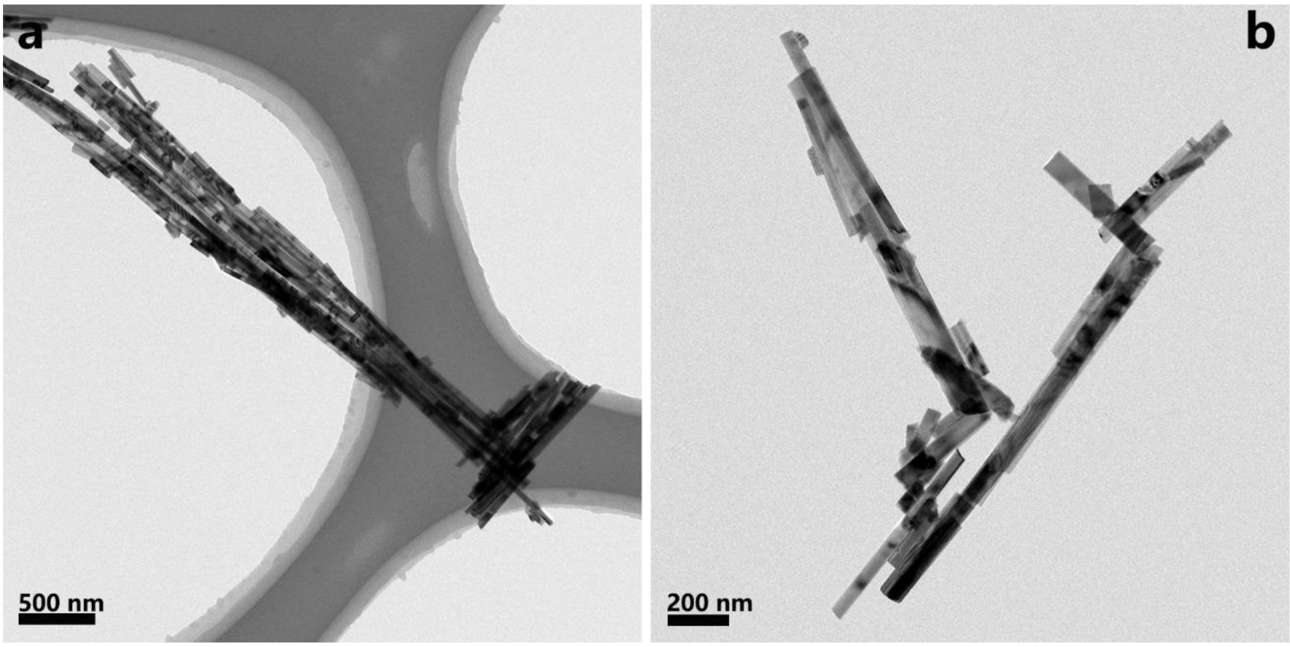


**Supplementary Fig. 6** TEM images of CdS@PEI10.

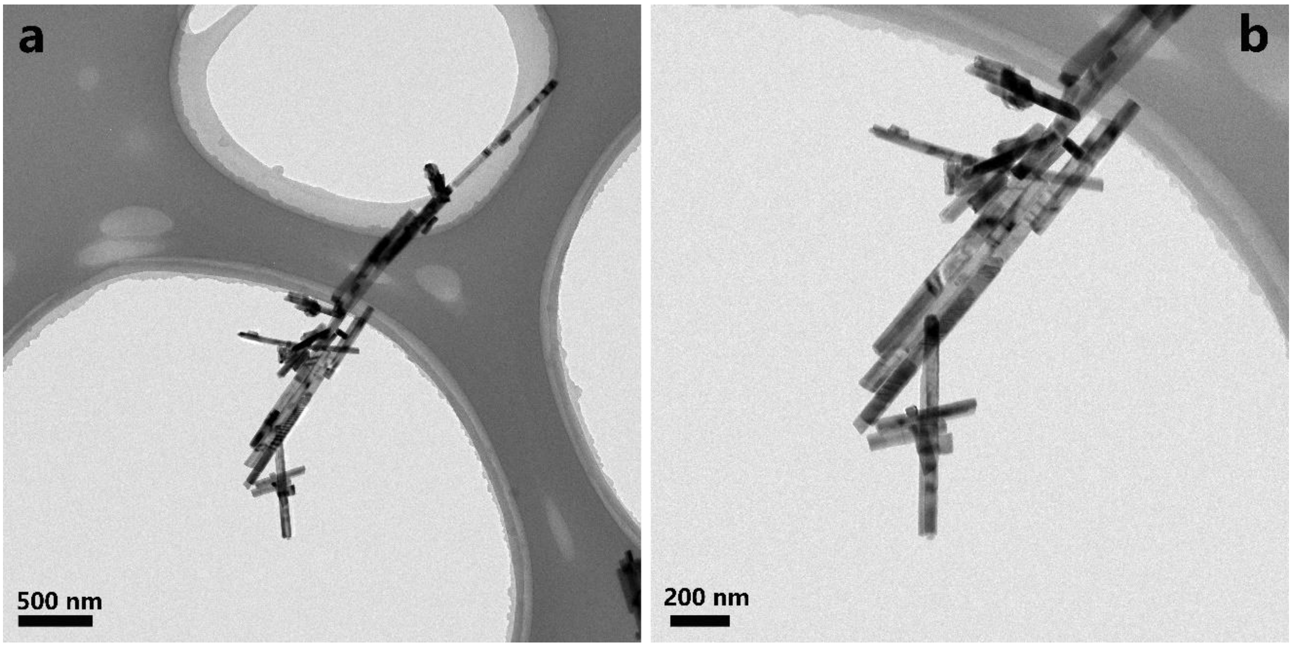


**Supplementary Fig. 7** TEM images of CdS@PAH7.

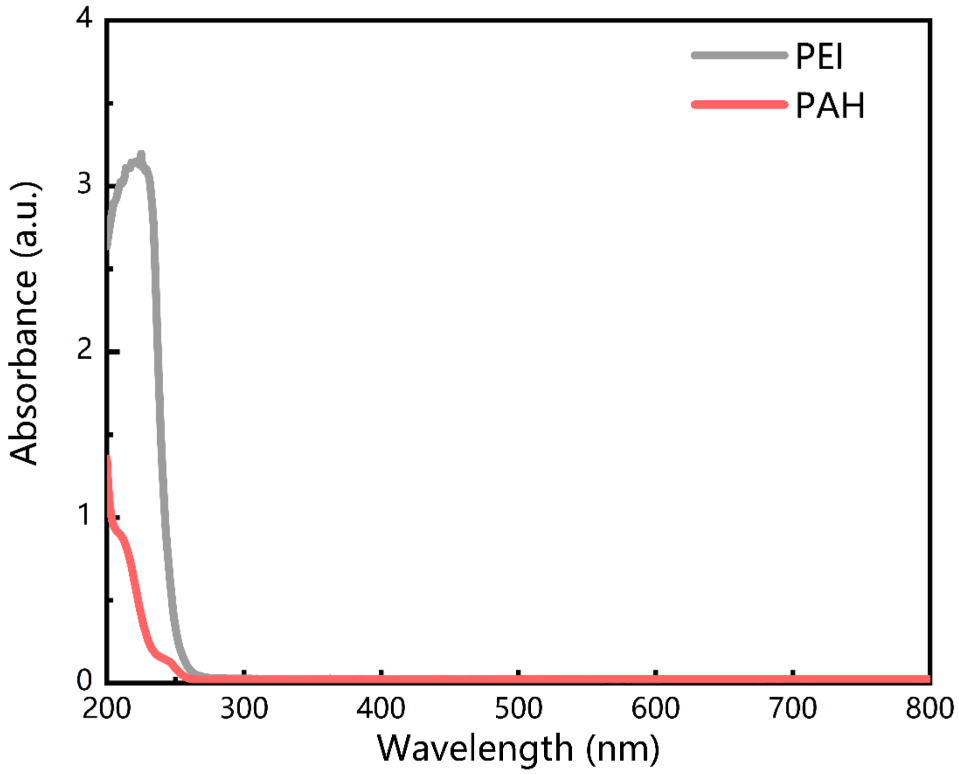


**Supplementary Fig. 8** UV-vis absorption spectrum of PEI and PAH aqueous solution.

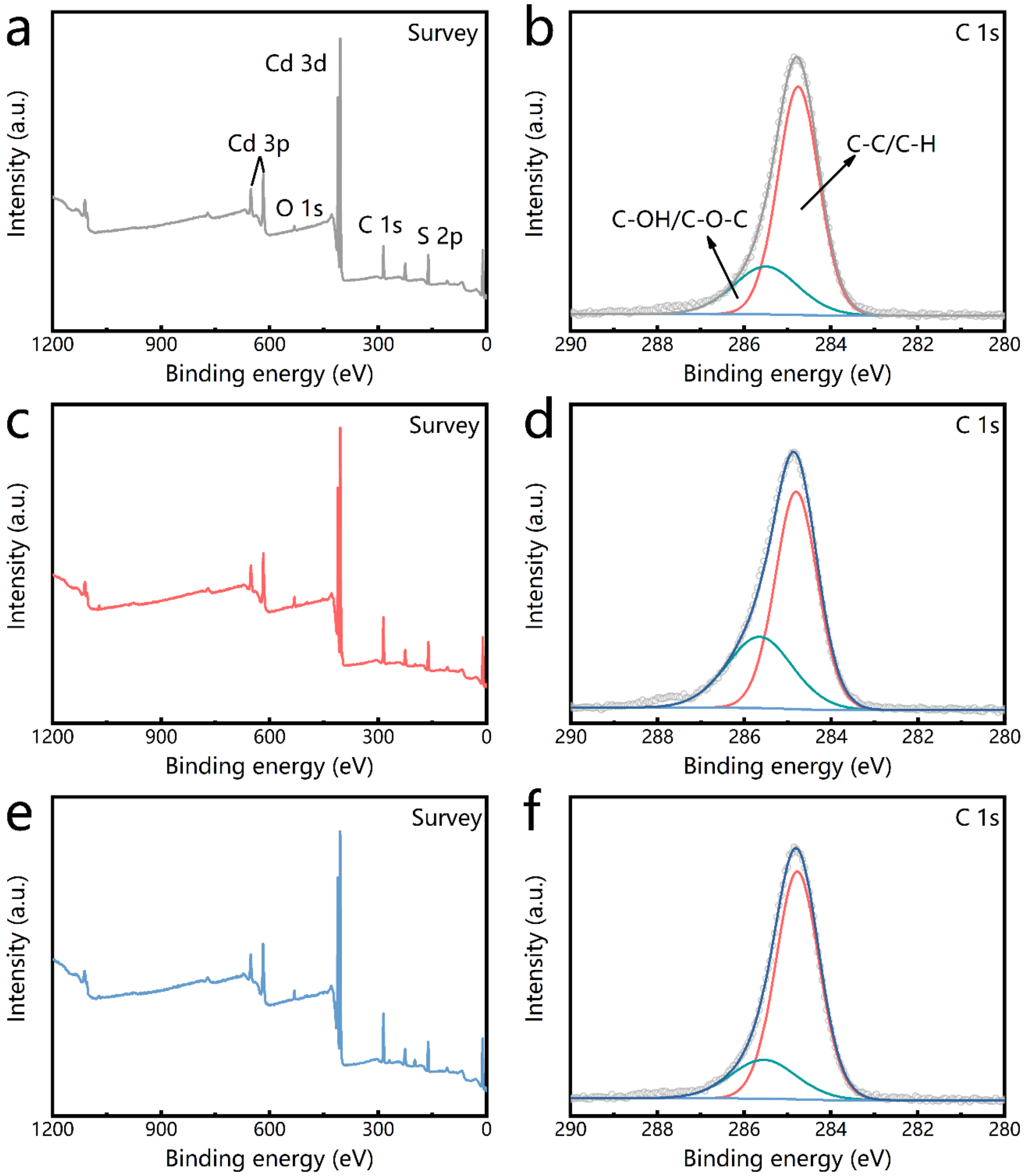


**Supplementary Fig. 9** Survey spectra and high-resolution C 1s spectrum of **a** & **b** CdS NWs, **c** & **d** CdS@PEI10, and **e** & **f** CdS@PAH7.

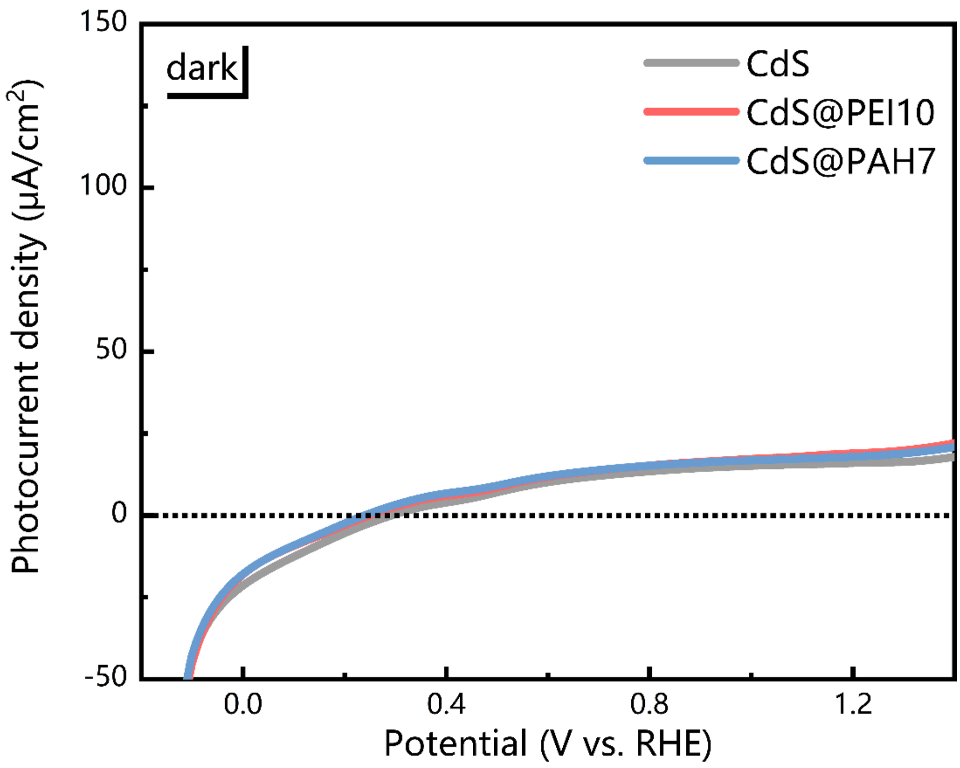


**Supplementary Fig. 10** LSV (scan rate 5 mV $s^{-1}$) of CdS NWs, CdS@PEI10, and CdS@PAH7 in dark.

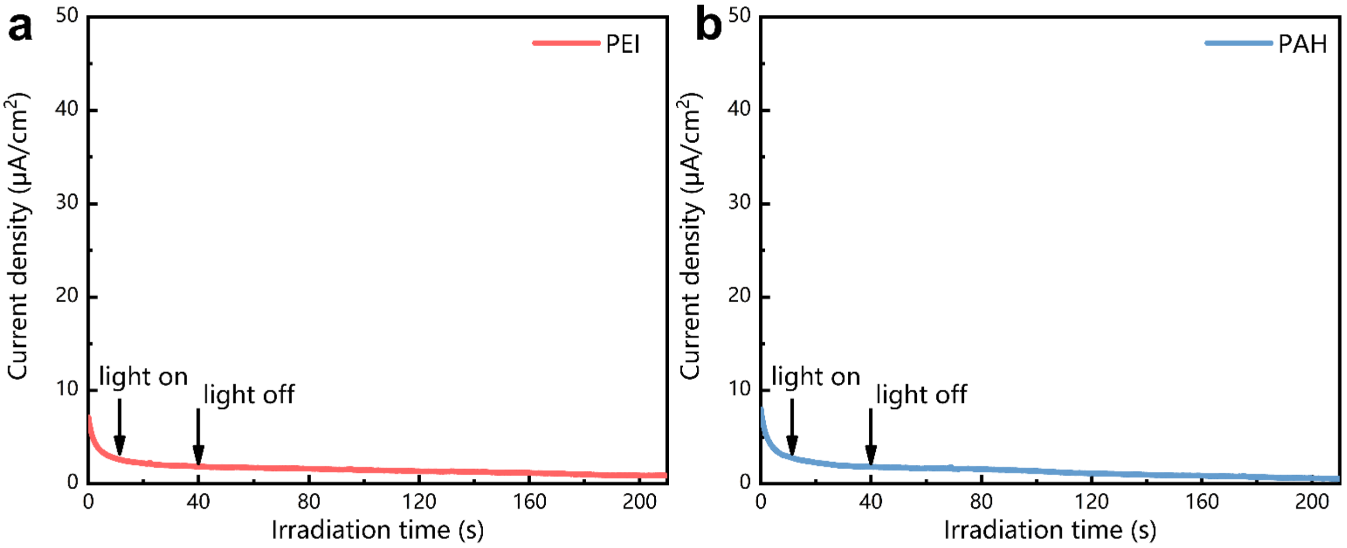


**Supplementary Fig. 11** I-t (bias: 1.2 V vs. RHE) of pure PEI and PAH in dark.

Conductivity of PEI and PAH can be evaluated by EIS results in dark, wherein generic charge transfer capability of the electrodes is probed by the variation of semicircular arc radius.[7,8] As displayed in **Supplementary Fig. 12**, the semicircular arc radius of the EIS curves for the blank CdS, CdS@PEI10, and CdS@PAH7 electrodes were probed in dark, which shows that CdS@PEI10 and CdS@PAH7 have larger semicircle arc radius than blank CdS. Apparently, interfacial charge transfer resistance of the electrodes increases after PEI/PAH encapsulation, which manifests that solid-state ultrathin PEI or PAH layer coated on the surface of semiconductor serves as an insulating polymer layer in our system.

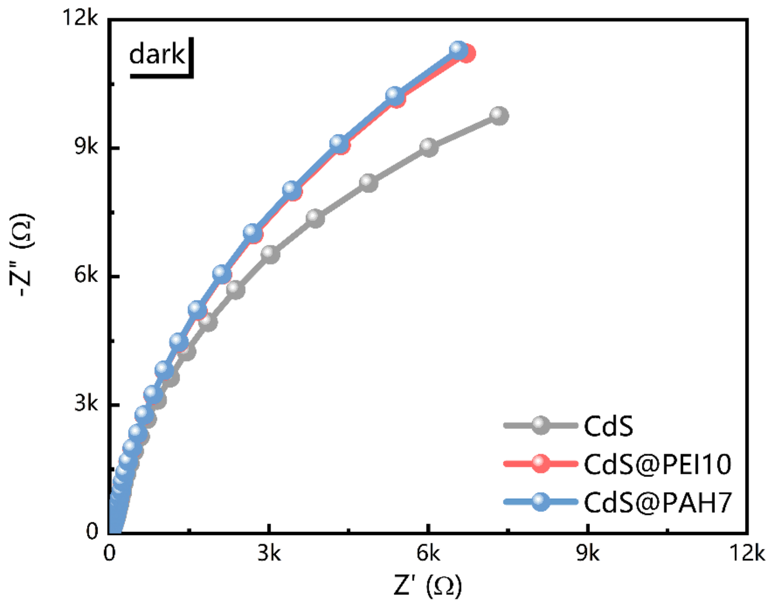


**Supplementary Fig. 12** EIS Nyquist plots of CdS, CdS@PEI10, and CdS@PAH7 in dark.

**Supplementary Fig. 13** shows the temporal evolution of the spectral changes during the photocatalytic reduction of 4-NA over CdS@PEI10 under visible light. There is a rapid decrease in the absorption of 4-NA at 380 nm along with the simultaneous appearance of two peaks at 238 and 302 nm. The peaks observed at 238 and 302 nm are the characteristic peaks of 4-PDA, suggesting progressive conversion of 4-NA to 4-PDA under visible light irradiation. Moreover, the isosbestic point was observed at 260 nm, exhibiting the complete transformation of 4-NA to 4-PDA without side reaction.[9]

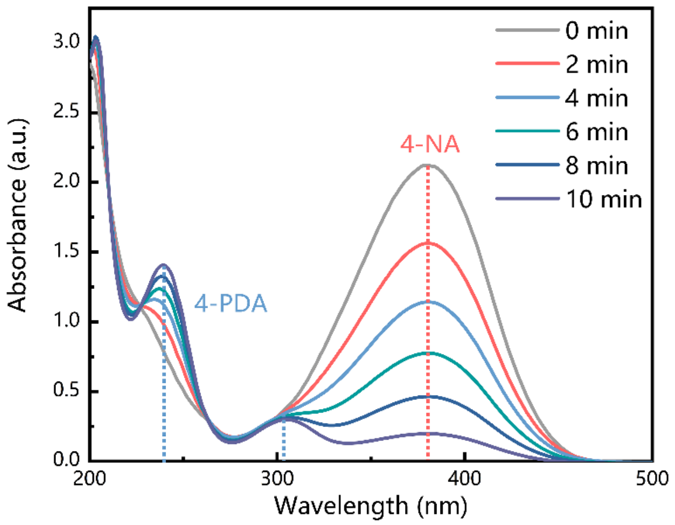


**Supplementary Fig. 13** UV-vis absorption spectra of 4-NA were collected after designated irradiation time (2 min) when it was photoreduced over CdS@PEI10 under visible light irradiation with the addition of ammonium formate as hole quencher and $N_2$ purge under ambient conditions.

Blank experiments (without light or catalysts) under identical experimental conditions demonstrate negligible photoactivities, suggesting the reaction is truly driven by a photocatalytic process rather than a physical adsorption process. Moreover, to deeply understand the decisive role of photogenerated electrons in triggering the photocatalytic selective reduction of 4-NA over CdS@NCPs, a control experiment with the addition of $K_2S_2O_8$ as electron scavenger for quenching photoelectrons was carried out. As can be seen from **Supplementary Fig. 14**, photocatalytic selective reduction of 4-NA over CdS@PEI10 and CdS@PAH7 is remarkably retarded when $K_2S_2O_8$ is added into the reaction system, persuasively highlighting the crucial role of photoelectrons in this photoreduction reaction. Considering that the direct addition of PEI or PAH into the reaction system could not readily boost the photoreduction activity of CdS NWs, it can be concluded that the systematic interaction between CdS NWs and PEI/PAH layer plays a crucial role in the significantly enhanced photocatalytic activity.

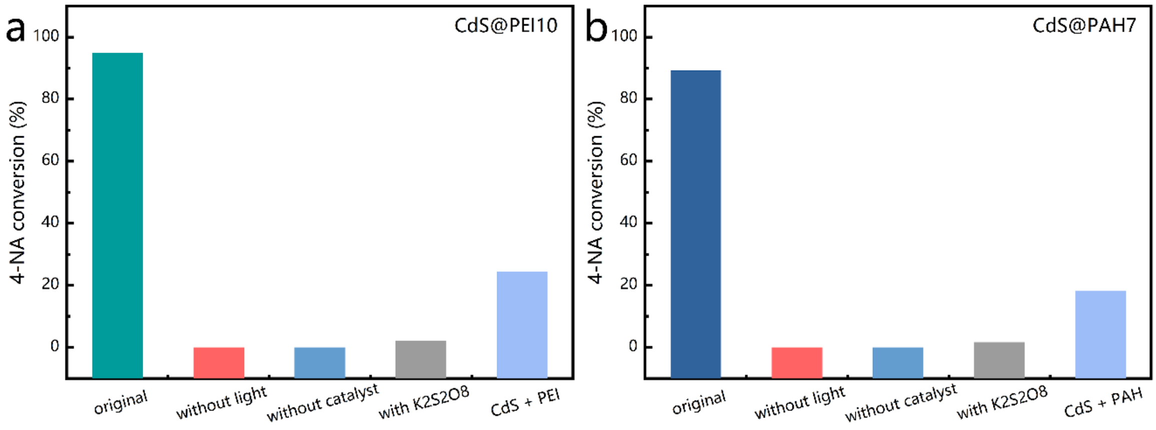


**Supplementary Fig. 14** Blank experiments for photocatalytic reduction of 4-NA without light irradiation or without adding photocatalyst, control experiments with and without adding $K_2S_2O_8$ as an electron scavenger, and photoactivity of CdS NWs toward photoreduction of 4-NA by adding PEI or PAH directly to the reaction system.

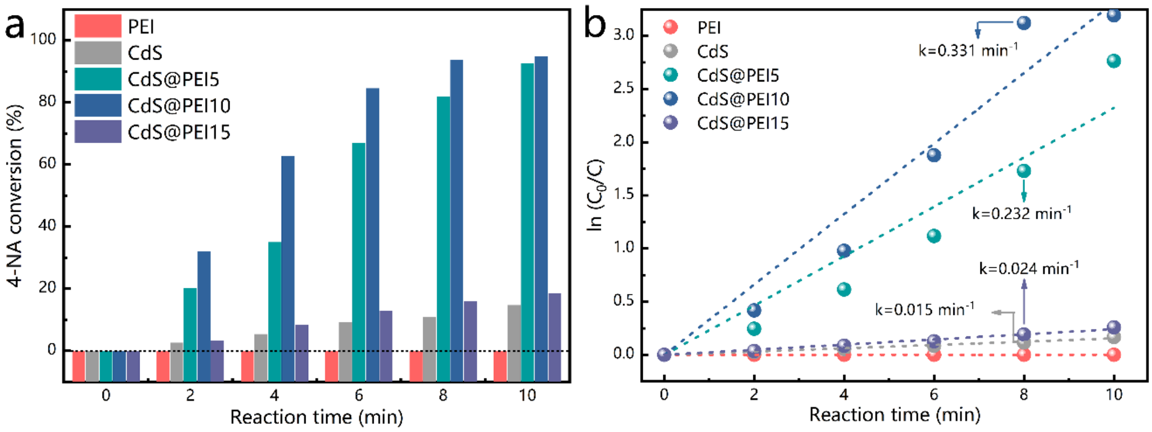


**Supplementary Fig. 15 a** Photocatalytic performances of pure CdS NWs and CdS@PEI with different concentration capping of PEI solution toward selective reduction of 4-NA to 4-PDA under visible light irradiation with the corresponding **b** kinetic curves.

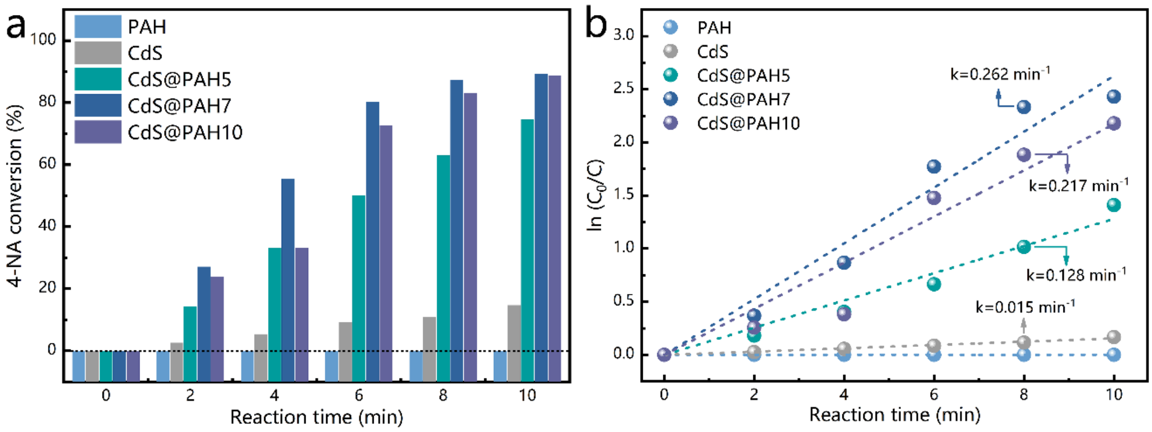


**Supplementary Fig. 16 a** Photocatalytic performances of pure CdS NWs and CdS@PAH with different concentration capping of PAH solution toward selective reduction of 4-NA to 4-PDA under visible light irradiation with the corresponding **b** kinetic curves.

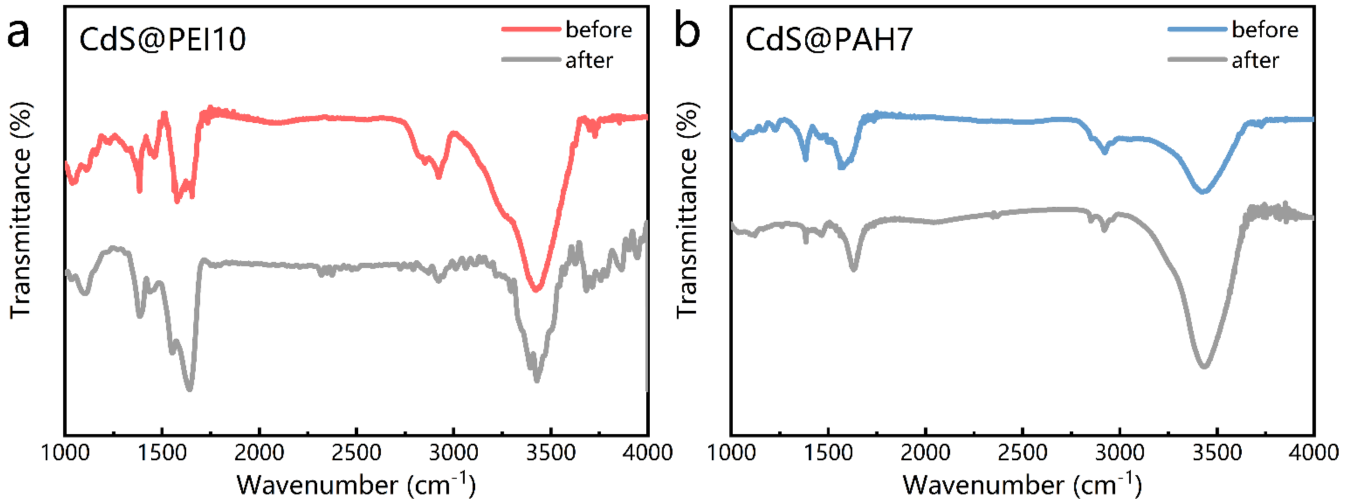


**Supplementary Fig. 17** FTIR spectra of **a** CdS@PEI10 and **b** CdS@PAH7 before and after cyclic 4-NA photoreduction.

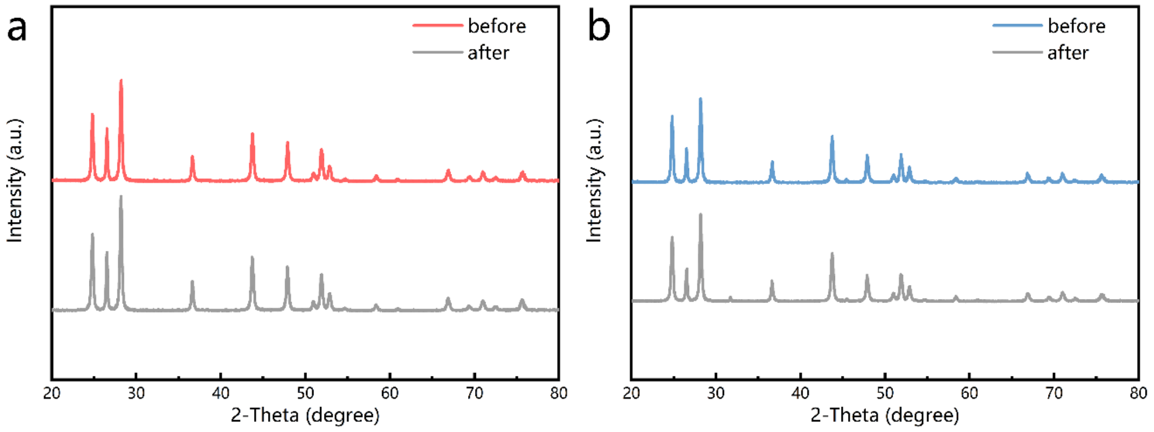


**Supplementary Fig. 18** XRD patterns of **a** CdS@PEI10 and **b** CdS@PAH7 before and after cyclic 4-NA photoreduction.

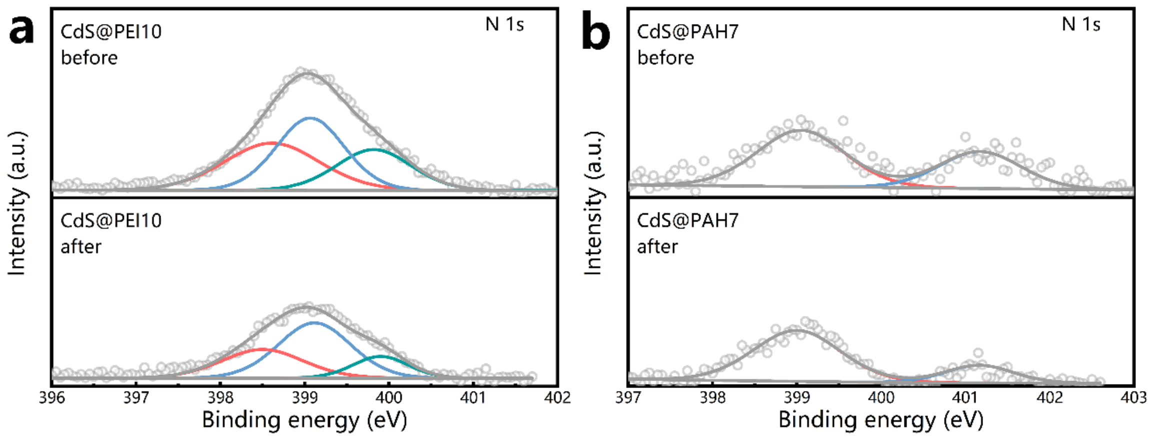


**Supplementary Fig. 19** High-resolution N 1s of **a** CdS@PEI10 and **b** CdS@PAH7 before and after cyclic 4-NA photoreduction.

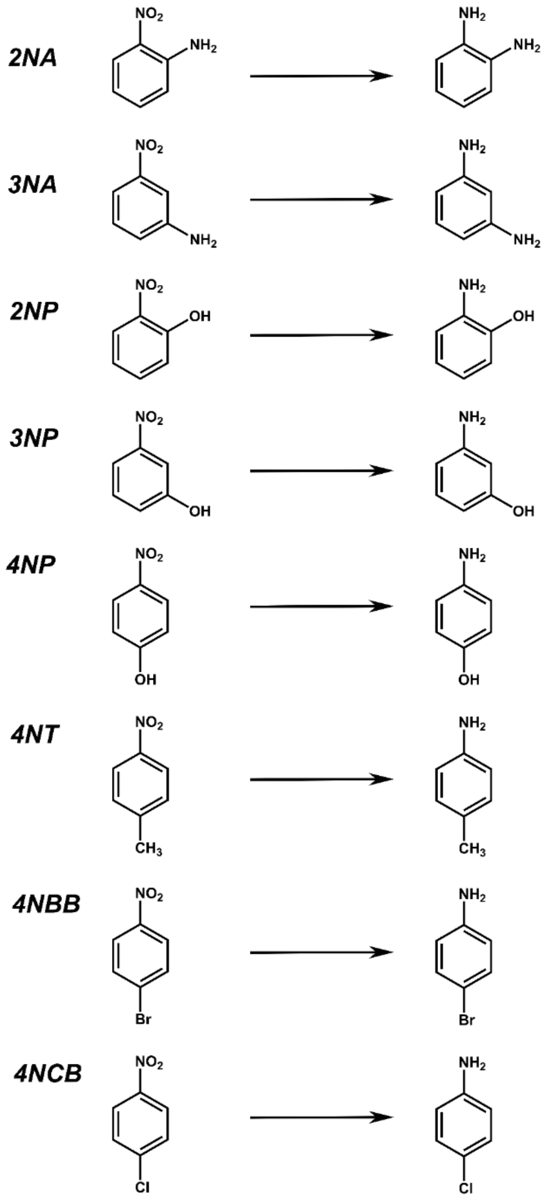


**Supplementary Fig. 20** Photoreduction reaction chemical formulas of aromatic nitro compounds.

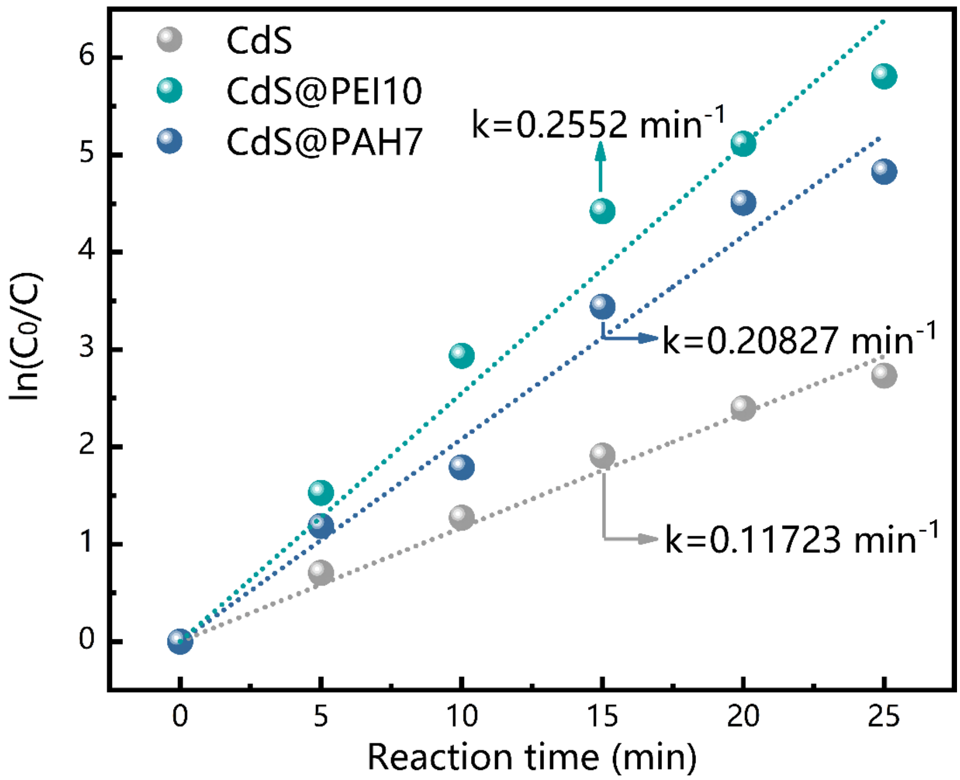


**Supplementary Fig. 21** Kinetic curves of CdS NWs, CdS@PEI10, and CdS@PAH7 toward photocatalytic mineralization of RhB under visible light irradiation.

$SiO_2$ layer functions as an ultra-thin insulating layer to inhibit the charge transfer between CdS NWs and PEI/PAH and the result proves PEI and PAH can relay electrons.

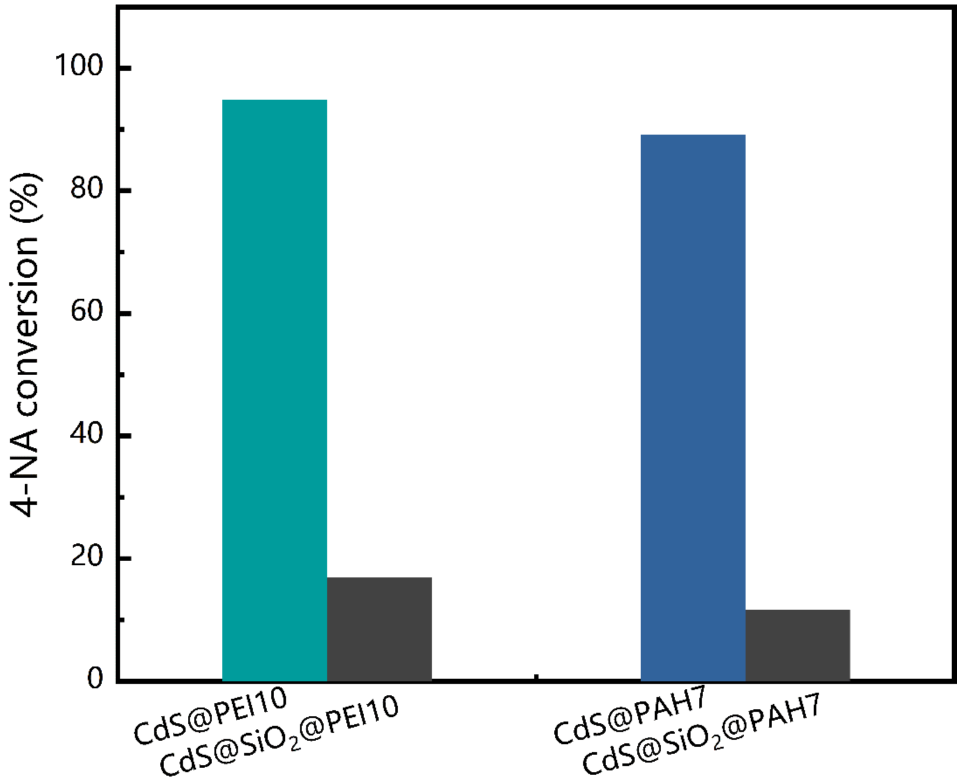


**Supplementary Fig. 22** Photoactivities of CdS@PEI10, CdS@$SiO_2$@PEI10, CdS@PAH7, and CdS@$SiO_2$@PAH7 toward selective reduction of 4-NA.

**Supplementary Table 1** Peak position with corresponding functional groups for all samples.

| *Peak position (cm$^{-1}$)* | *CdS NWs* | *CdS@PEI10* | *CdS@PAH7* | *Reference* |
|---|---|---|---|---|
| 3436 | $\nu_{O-H}$ | $\nu_{O-H}$ | $\nu_{O-H}$ | 10 |
| 2929 | $\nu_{C-H}$ | $\nu_{C-H}$ | $\nu_{C-H}$ | 11,12 |
| 2854 | $\nu_{C-H}$ | $\nu_{C-H}$ | $\nu_{C-H}$ | 11,12 |
| 1631 | $\delta_{O-H}$ | $\delta_{O-H}$ | $\delta_{O-H}$ | 10 |
| 1584 | N.D. | $\delta_{N-H}$ | $\delta_{N-H}$ | 13,14 |
| 1384 | $\delta_{C-H}$ | $\delta_{C-H}$ | $\delta_{C-H}$ | 11,12 |
| 1037 | $\nu_{C-O-C}$ | $\nu_{C-O-C}$ | $\nu_{C-O-C}$ | 10 |

N.D.: Not Detected.

ν: Stretching vibration.

δ: Deformation vibration

**Supplementary Table 2** Chemical bond species vs. B.E. for all samples.

| *Element ($cm^{-1}$)* | *PEI* | *PAH* | *CdS NWs* | *CdS@PEI10* | *CdS@PAH7* | *Species* |
|---|---|---|---|---|---|---|
| C 1s A | 284.8 | 284.8 | 284.8 | 284.8 | 284.8 | C-C/C-H |
| C 1s B | 285.5 | 285.5 | 285.5 | 285.5 | 285.5 | -COO [15] |
| N 1s A | 398.1 | N.D. | N.D. | 398.6 | N.D. | -NH- [16] |
| N 1s B | 398.5 | N.D. | N.D. | 399.0 | N.D. | $-NH_2$ [16] |
| N 1s C | N.D. | N.D. | N.D. | 399.9 | N.D. | -CONH [17] |
| N 1s D | N.D. | 401.3 | N.D. | N.D. | 401.0 | $-NH_3^+$ [18] |
| N 1s E | N.D. | 399.0 | N.D. | N.D. | 399.0 | adsorbed N |
| Cd $3d_{5/2}$ | N.D. | N.D. | 404.4 | 404.3 | 404.6 | $Cd^{2+}$ [19] |
| Cd $3d_{3/2}$ | N.D. | N.D. | 411.1 | 411 | 411.3 | $Cd^{2+}$ [19] |
| S $2p_{3/2}$ | N.D. | N.D. | 160.8 | 160.6 | 161.0 | $S^{2-}$ [20] |
| S $2p_{1/2}$ | N.D. | N.D. | 162.1 | 161.8 | 162.2 | $S^{2-}$ [20] |

N.D.: Not Detected.

Equivalent circuit used for fitting the EIS data is composed of a series of resistance, wherein $R_s$ is the solution resistance, CPE refers to the constant phase element, and $R_{ct}$ indicates the charge transfer resistance at the electrode/electrolyte interface.[21] In general, the impedance of a CPE is described following:

$$Z = (CPET)^{-1}(i\omega)^{-CPEP}$$

where CPE-T represents the capacitance value, ω is the angular frequency, and CPE-P is the ideality factor.

**Supplementary Table 3** Fitted EIS results of electrodes in dark based on the equivalent circuit.

| Photoanode | $R_s$ (ohm) | $R_{ct}$ (ohm) | CPE-T | CPE-P |
|---|---|---|---|---|
| CdS NWs | 7.231 | 83619 | 12.127 | 0.79027 |
| CdS@PEI10 | 7.105 | 186180 | 11.618 | 0.8012 |
| CdS@PAH7 | 7.103 | 226630 | 11.630 | 0.79941 |

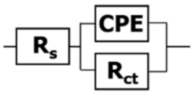


**Supplementary Table 4** Fitted EIS results of electrodes under visible light irradiation conditions based on the equivalent circuit.

| Photoanode | $R_s$ (ohm) | $R_{ct}$ (ohm) | CPE-T | CPE-P |
|---|---|---|---|---|
| CdS NWs | 7.239 | 63509 | 12.559 | 0.78937 |
| CdS@PEI10 | 7.965 | 18229 | 28.297 | 0.84203 |
| CdS@PAH7 | 7.826 | 23433 | 28.145 | 0.8434 |

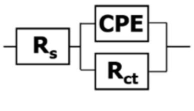

**Supplementary Table 5** Conversion of different samples toward selective photoreduction of 4-NA to 4-PDA under visible light irradiation for 10 min.

| ***Sample*** | ***Conversion (%)*** |
|---|---|
| PEI | 0 |
| PAH | 0 |
| CdS NWs | 14.7 |
| CdS@PEI5 | 92.7 |
| CdS@PEI10 | 94.9 |
| CdS@PEI15 | 18.5 |
| CdS@PAH5 | 74.6 |
| CdS@PAH7 | 89.2 |
| CdS@PAH9 | 88.7 |

**Supplementary Table 6** Comparison of 4-NA conversion for this work with the reported CdS-based photocatalysts.

| ***Number*** | ***Materials*** | ***Synthetic method*** | ***Light source*** | ***Conversion*** | ***Reference*** |
|---|---|---|---|---|---|
| This work | CdS@PEI10<br>CdS@PAH7 | Self-assembly | Visible light | 94.9%<br>89.2% within 10 min | / |
| 1 | CQD-CdS | Mechanical mixing | Visible light | ca. 30% within 6 min | 22 |
| 2 | CdS-$BiVO_4$ | Self-assembly | Visible light | 99% within 90 min | 23 |
| 3 | CdS/$TiO_2$ | Hydrothermal | Visible light | ca. 45% within 4 h | 24 |
| 4 | CdS@$ZnIn_2S_4$ | Hydrothermal | Visible light | ca. 99% within 160 min | 25 |
| 5 | C dot/CdS | Self-assembly | Visible light | 70% within 150 min | 26 |
| 6 | CdS/ZnO/GO | Refluxing | Visible light | 95% within 16 min | 27 |
| 7 | ZnO-Au@CdS | Photodeposition | Visible light | 92% within 14 min | 28 |
| 8 | CdS-$MoS_2$/rGO | Hydrothermal | Visible light | 100% within 20 min | 29 |
| 9 | CdS/$In_2O_3$ | Photodeposition | 400-800 nm | 80% within 30 min | 30 |
| 10 | CdS-MIL68 | Photodeposition | Visible light | 100% within 8 min | 31 |

# Supplementary References


1 Han, C., Quan, Q., Chen, H. M., Sun, Y. & Xu, Y.-J. Progressive Design of Plasmonic Metal–Semiconductor Ensemble toward Regulated Charge Flow and Improved Vis–NIR-Driven Solar-to-Chemical Conversion. *Small* **13**, 1602947 (2017).

2 Chen, D. *et al.* Synthesis of Monodisperse Mesoporous Titania Beads with Controllable Diameter, High Surface Areas, and Variable Pore Diameters (14−23 nm). *J. Am. Chem. Soc.* **132**, 4438-4444 (2010).

3 Li, Y.-Y. *et al.* Selective Photocatalytic Reduction of CO2 to CH4 Modulated by Chloride Modification on $Bi_2WO_6$ Nanosheets. *ACS Appl. Mater. Interfaces* **12**, 54507-54516 (2020).

4 Segall, M. D. *et al.* First-principles simulation: ideas, illustrations and the CASTEP code. *J. Phys.: Condens. Matter* **14**, 2717-2744 (2002).

5 Perdew, J. P., Burke, K. & Ernzerhof, M. Generalized Gradient Approximation Made Simple. *Phys. Rev. Lett.* **77**, 3865-3868 (1996).

6 Grimme, S. Accurate description of van der Waals complexes by density functional theory including empirical corrections. *J. Comput. Chem.* **25**, 1463-1473 (2004).

7 Li, N. *et al.* Battery Performance and Photocatalytic Activity of Mesoporous Anatase $TiO_2$ Nanospheres/Graphene Composites by Template-Free Self-Assembly. *Adv. Funct. Mater.* **21**, 1717-1722 (2011).

8 Fu, Y. *et al.* Phase-Modulated Band Alignment in CdS Nanorod/$SnS_x$ Nanosheet Hierarchical Heterojunctions toward Efficient Water Splitting. *Adv. Funct. Mater.* **28**, 1706785 (2018).

9 Liu, S. & Xu, Y.-J. Efficient electrostatic self-assembly of one-dimensional CdS–Au nanocomposites with enhanced photoactivity, not the surface plasmon resonance effect. *Nanoscale* **5**, 9330-9339 (2013).

10 Roy, J. S., Pal Majumder, T. & Schick, C. Optical characterization of CdS nanorods capped with starch. *J. Mol. Struct.* **1088**, 95-100 (2015).

11 Müller, M. *et al.* An in-situ ATR-FTIR study on polyelectrolyte multilayer assemblies on solid surfaces and their susceptibility to fouling. *Macromol. Rapid Commun.* **19**, 333-336 (1998).

12 Slika, L., Moubarak, A., Borjac, J., Baydoun, E. & Patra, D. Preparation of curcumin-poly (allyl amine) hydrochloride based nanocapsules: Piperine in nanocapsules accelerates encapsulation and release of curcumin and effectiveness against colon cancer cells. *Mater. Sci. Eng. C* **109**, 110550 (2020).

13 Ananth, A., Arthanareeswaran, G. & Wang, H. The influence of tetraethylorthosilicate and polyethyleneimine on the performance of polyethersulfone membranes. *Desalination* **287**, 61-70 (2012).

14 Zucolotto, V. *et al.* Unusual Interactions Binding Iron Tetrasulfonated Phthalocyanine and Poly(allylamine hydrochloride) in Layer-by-Layer Films. *J. Phys. Chem. B* **107**, 3733-3737 (2003).

15 Fujimoto, A., Yamada, Y., Koinuma, M. & Sato, S. Origins of $sp^3$C peaks in $C_{1s}$ X-ray Photoelectron Spectra of Carbon Materials. *Anal. Chem.* **88**, 6110-6114 (2016).

16 Wu, W.-C. *et al.* Nitrogen-doped carbon nanodots prepared from polyethylenimine for fluorometric determination of salivary uric acid. *Microchim. Acta* **186**, 166 (2019).

17 Jansen, R. J. J. & van Bekkum, H. XPS of nitrogen-containing functional groups on activated carbon. *Carbon* **33**, 1021-1027 (1995).

18 Lourenço, J. M. C. *et al.* Counterions in Poly(allylamine hydrochloride) and Poly(styrene sulfonate) Layer-by-Layer Films. *Langmuir* **20**, 8103-8109 (2004).

19 Sun, Z., Chen, H., Zhang, L., Lu, D. & Du, P. Enhanced photocatalytic $H_2$ production on cadmium sulfide photocatalysts using nickel nitride as a novel cocatalyst. *J. Mater. Chem. A* **4**, 13289-13295 (2016).

20 Zhong, Y., Zhao, G., Ma, F., Wu, Y. & Hao, X. Utilizing photocorrosion-recrystallization to prepare a highly stable and efficient CdS/$WS_2$ nanocomposite photocatalyst for hydrogen evolution. *Appl. Catal. B* **199**, 466-472 (2016).

21 Xiao, Y.-H. & Zhang, W.-D. $MoS_2$ quantum dots interspersed $WO_3$ nanoplatelet arrays with enhanced photoelectrochemical activity. *Electrochim. Acta* **252**, 416-423 (2017).

22 Chai, Y.-Y., Qu, D.-P., Ma, D.-K., Chen, W. & Huang, S. Carbon quantum dots/$Zn^{2+}$ ions doped-CdS nanowires with enhanced photocatalytic activity for reduction of 4-nitroaniline to p-phenylenediamine. *Appl. Surf. Sci.* **450**, 1-8 (2018).

23 Han, B., Liu, S., Xu, Y.-J. & Tang, Z.-R. 1D CdS nanowire–2D $BiVO_4$ nanosheet heterostructures toward photocatalytic

selective fine-chemical synthesis. *RSC Adv.* **5**, 16476-16483 (2015).

24 Hu, Z., Quan, H., Chen, Z., Shao, Y. & Li, D. New insight into an efficient visible light-driven photocatalytic organic transformation over CdS/$TiO_2$ photocatalysts. *Photochem. Photobiol. Sci.* **17**, 51-59 (2018).

25 Chen, W. *et al.* Hydrothermal route to synthesize helical CdS@$ZnIn_2S_4$ core-shell heterostructures with enhanced photocatalytic hydrogeneration activity. *Ceram. Int.* **45**, 1803-1811 (2019).

26 Chai, N.-N., Wang, H.-X., Hu, C.-X., Wang, Q. & Zhang, H.-L. Well-controlled layer-by-layer assembly of carbon dot/CdS heterojunctions for efficient visible-light-driven photocatalysis. *J. Mater. Chem. A* **3**, 16613-16620 (2015).

27 Han, C., Chen, Z., Zhang, N., Colmenares, J. C. & Xu, Y.-J. Hierarchically CdS Decorated 1D ZnO Nanorods-2D Graphene Hybrids: Low Temperature Synthesis and Enhanced Photocatalytic Performance. *Adv. Funct. Mater.* **25**, 221-229 (2015).

28 Zhang, N., Xie, S., Weng, B. & Xu, Y.-J. Vertically aligned ZnO–Au@CdS core–shell nanorod arrays as an all-solid-state vectorial Z-scheme system for photocatalytic application. *J. Mater. Chem. A* **4**, 18804-18814 (2016).

29 Peng, W.-c., Chen, Y. & Li, X.-y. $MoS_2$/reduced graphene oxide hybrid with CdS nanoparticles as a visible light-driven photocatalyst for the reduction of 4-nitrophenol. *J. Hazard. Mater.* **309**, 173-179 (2016).

30 Li, X., Wang, C., Li, B., Shao, Y. & Li, D. Efficient light harvesting over a CdS/$In_2O_3$ photonic crystal photocatalyst for hydrogenation of 4-nitroaniline to p-phenylenediamine. *Phys. Chem. Chem. Phys.* **18**, 27848-27857 (2016).

31 Liang, R., Jing, F., Yan, G. & Wu, L. Synthesis of CdS-decorated MIL-68(Fe) nanocomposites: Efficient and stable visible light photocatalysts for the selective reduction of 4-nitroaniline to p-phenylenediamine in water. *Appl. Catal. B* **218**, 452-459 (2017).
