# Supplementary material for: Accelerating charge transfer via nonconjugated polyelectrolyte interlayers toward efficient versatile photoredox catalysis
Source: Commun Chem. 2021 Oct 22;4:150. doi: 10.1038/s42004-021-00589-w (PMC9814354; doi:10.1038/s42004-021-00589-w)
Supplement: Supplementary file 2 — Description of Additional Supplementary Files [file 42004_2021_589_MOESM2_ESM.pdf]

Description of Additional Supplementary Files

File Name: Supplementary Data 1

Description: The most stable fractional coordinates for PEI/PAH adsorbates on CdS (110) surface.
